# (*E*)-Nerolidol is a volatile signal that induces defenses against insects and pathogens in tea plants

**DOI:** 10.1038/s41438-020-0275-7

**Published:** 2020-04-01

**Authors:** Shenglong Chen, Liping Zhang, Xiaoming Cai, Xin Li, Lei Bian, Zongxiu Luo, Zhaoqun Li, Zongmao Chen, Zhaojun Xin

**Affiliations:** 1 Tea Research Institute, Chinese Academy of Agricultural Sciences, Hangzhou, 310008 China; 2 Key Laboratory of Tea Biology and Resources Utilization, Ministry of Agriculture, Hangzhou, 310008 China

**Keywords:** Plant immunity, Plant biotechnology

## Abstract

Plants release large amounts of volatile organic compounds (VOCs) in response to attackers. Several VOCs can serve as volatile signals to elicit defense responses in undamaged tissues and neighboring plants, but many questions about the ecological functions of VOCs remain unanswered. Tea plants are impacted by two harmful invaders, the piercing herbivore *Empoasca* (*Matsumurasca*) *onukii* Matsuda and the pathogen *Colletotrichum fructicola*. To determine the VOC signals in tea, we confirmed CsOPR3 as a marker gene and set up a rapid screening method based on a 1.51 kb *CsOPR3* promoter fused with a β-glucuronidase (GUS) reporter construct (*OPR3p::GUS*) in *Arabidopsis*. Using this screening system, a terpenoid volatile (*E*)-nerolidol was identified as a potent signal that elicits plant defenses. The early responses triggered by (*E*)-nerolidol included the activation of a mitogen-activated protein kinase and WRKY, an H_2_O_2_ burst, and the induction of jasmonic acid and abscisic acid signaling. The induced plants accumulated high levels of defense-related chemicals, which possessed broad-spectrum anti-herbivore or anti-pathogen properties, and ultimately triggered resistance against *Empoasca onukii* and *Colletotrichum fructicola* in tea. We propose that these findings can supply an environmentally friendly management strategy for controlling an insect pest and a disease of tea plants.

## Introduction

Green plants are susceptible to pervasive attack by herbivorous insects and microbial pathogens during their life cycle. To effectively combat attacks by these invaders, plants have evolved a diverse array of defense mechanisms, which include preexisting physical barriers and inducible defense responses that are activated upon attack^1^. The induced defense mechanisms are usually associated with reactive oxygen species (ROS)^2^, mitogen-activated protein kinase (MAPK) cascades^3^ and signaling modulated by phytohormones, such as jasmonic acid (JA), salicylic acid (SA), abscisic acid (ABA), and ethylene (ET). The reprogramming of metabolic pathways ultimately triggers the secretion of plant secondary metabolites as defenses against herbivores and pathogens^1,4,5^. The defensive strategies may include indirect mechanisms in which herbivore-challenged plants emit volatile organic compounds (VOCs) to recruit parasitoids and natural enemies of insects and bolster resistance to future threats^6^. The pathogen-infested plants also release unique blends of VOCs to inhibit pathogen growth directly or promote plant resistance/susceptibility to pathogen attack^7^. In contrast, direct defense mechanisms include the use of toxic metabolites such as phytoalexins and protease inhibitors, physical barriers such as callose and lignin, and other nonvolatile defense-related enzymes such as phenylalanine ammonia lyase (PAL) and polyphenol oxidases (PPOs) which are stored in specialized cells to be activated when plants are attacked by pathogens or herbivores^4^. To activate these different defenses, distinct defense pathways are involved in the response to insects and pathogens in plants. The SA and H_2_O_2_ pathways mainly induce resistance against biotrophic pathogens and most sucking/piercing insects, whereas the JA pathway mainly confers resistance against necrotrophic pathogens, several phloem-sap-sucking insects and chewing herbivores^8,9^.

The exogenous application of naturally occurring phytohormones in the defense signaling pathway, and chemical elicitors that are not found in plants can elicit plant defense responses similar to those induced by herbivores or pathogens^10^. Several chemical elicitors, such as β-aminobutyric acid, benzothiadiazole, 2,4-dichlorophenoxyacetic acid (2,4-D), and laminarin, are highly active in the induction of plant defense responses to herbivores and/or pathogens^11–13^. They can protect plants from a broad-spectrum of related diseases or pests and are useful tools for dissecting the molecular components of the plant immune system. Chemical elicitors can be deployed as an environmentally safe strategy for the control of harmful invaders.

The tea plant (*Camellia sinensis*) is an economically important woody crop in Asia. Tea is valuable for human health because of its beneficial metabolites^14^. Similar to other plants, tea plants suffer greatly from many pests and diseases throughout their life cycle. A serious pest of tea plants is the tea green leafhopper (TLH), *Empoasca* (*Matsumurasca*) *onukii* Matsuda (Hemiptera: Cicadellidae). TLH is a harmful species that feeds via cell rupture and usually produces ten generations every year^15^. Nymphs and adults of TLH suck cell sap from tender stems, young leaves, and buds, resulting in the yellowing, browning, and drying of the plant. Anthracnose, which is caused by *Colletotrichum*, is a serious disease of tea plants. The fungal pathogen *Colletotrichum fructicola* is one of the causal agents of the disease^16^. Symptoms on young leaves first appear as water-soaked lesions. The lesions expand over time and become necrotic as the disease progresses, leading to massive cell death and tissue destruction^16^. Thus, *C. fructicola* is a severe fungal pathogen that causes total yield losses in this crop. To date, the most common method used to manage tea pests and diseases has been the regular application of chemical insecticides and other pesticides. However, the massive use of pesticides poses serious threats to human health and environmental safety.

In response to attack by invaders, plants usually release increased amounts of VOCs, which include chemicals such as terpenoids, green leaf volatiles (GLV), nitrogenous compounds, and other aromatic compounds^17^. In tea plants, herbivore feeding can induce the release of over 50 kinds of volatiles^18^. In addition to protecting plants by attracting herbivore enemies, VOCs can serve as volatile signals to elicit defense responses in undamaged tissues or neighboring plants via the JA pathway^19^. To date, several individual components of VOCs, such as MeJA, 3E-4,8-dimethyl-l,3,7-nonatriene, ocimene, cis-jasmone, indole, and GLVs, have been shown to fortify plant defenses against a number of chewing herbivores^20,21^. However, the roles of these VOCs in modulating plant defense against sucking pests and pathogens and the corresponding molecular mechanisms are not well understood.

This study was primarily aimed at determining the volatile signals of tea plants from VOCs and explaining the corresponding molecular mechanisms. A rapid method was set up for screening because bioassays of each compound on a large scale are labor intensive. Given the importance of the JA pathway in controlling piercing-sucking insects and necrotrophic pathogens, we used JA signaling as a marker pathway. In plants, JA is produced from free linolenic acid, which is converted to 13S-hydroperoxyoctadecatrienoic acid (13S-HPOT) in a reaction catalyzed by lipoxygenase. 13S-HPOT is subsequently transformed into 12-oxo-phytodienoic acid (OPDA) under the action of allene oxide synthase and allene oxide cyclase in chloroplasts. Then, OPDA is transported to the peroxisome and reduced by OPDA reductase (OPR) to yield 3-oxo-2-(2-pentenyl)-cyclopentane-1-octanoic acid (OPC-8:0), followed by conversion to JA through β-oxidation^22^. Recently, we reported a putative OPR gene *CsOPR3* from herbivore-infested tea plants^23^. In the present study, we confirmed the function of *CsOPR3* in JA biosynthesis and identified it as a marker gene to rapidly screen volatile signals from VOCs according to their ability to induce defenses. We cloned a 1.5 kb promoter region of *CsOPR3* and fused the promoter to a β-glucuronidase (GUS) reporter gene (*OPR3p::GUS*). Using an *Agrobacterium*-based transformation system, we obtained T2 homozygous transgenic *Arabidopsis OPR3p::GUS* lines. Using the transgenic lines, we then identified (*E*)-nerolidol as a candidate volatile signal. (*E*)-Nerolidol could effectively activate MAPK and WRKY genes and increase JA, H_2_O_2_, and ABA production. Moreover, (*E*)-nerolidol induced the accumulation of defense-related compounds with extensive natural anti-herbivore or anti-pathogen effects, thereby enhancing the defense of tea plants against TLH and *C. fructicola*. Previous studies have paid more attention to the behavioral regulation function of (*E*)-nerolidol. Our results revealed for the first time that the molecular mechanism of (*E*)-nerolidol is to act as a volatile signal of herbivore/pathogen-resistance in tea plants. Our results may contribute to the development of novel approaches for the control of an important piercing pest and a pathogen.

## Results

### Wounding, JA treatment, and TLH infestation increase CsOPR3 accumulation

**Fig. 1:**
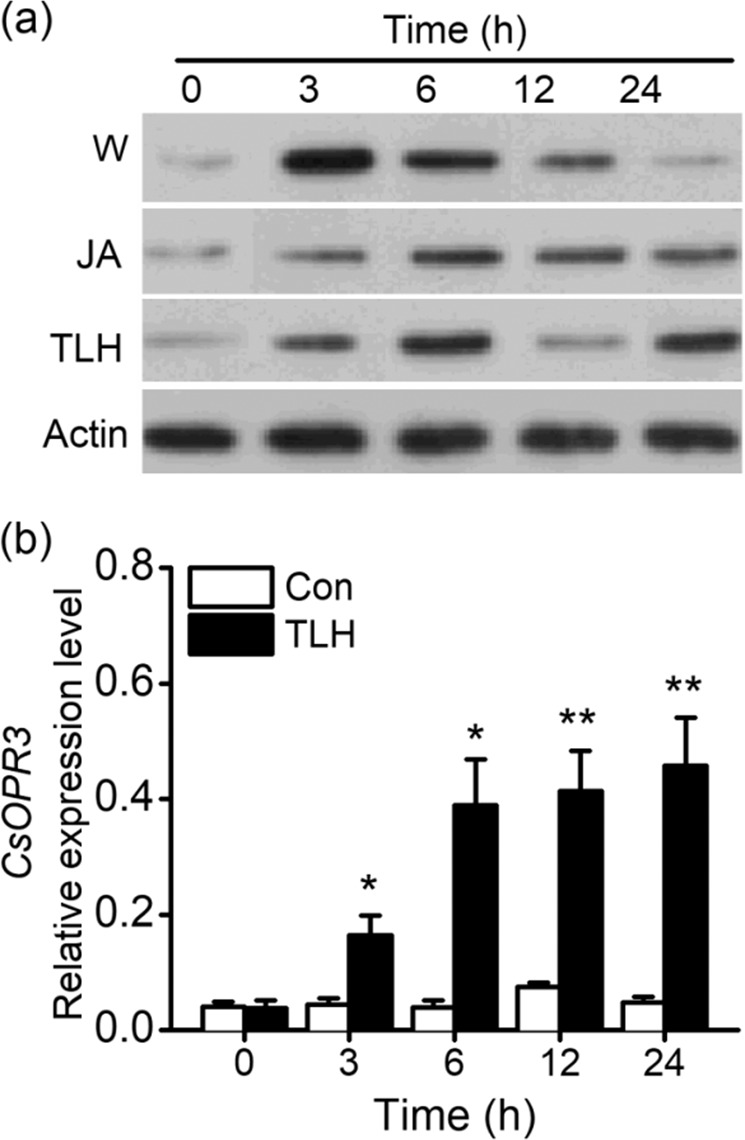
Protein and transcript levels of CsOPR3 in tea leaves under different treatments. **a** Western blot analysis of CsOPR3 accumulation in tea leaves subjected to the following treatments: mechanical wounding (W), jasmonic acid (JA, 150 μg ml^−1^), and *E. onukii* (TLH) infestation. **b** Relative expression level of *CsOPR3* in TLH-infested tea leaves and controls (Con). Values are means + SEs for five independent biological replicates. Asterisks indicate significant differences between TLH-treated and control plants (*t* test, *P < 0.05; **P < 0.01)

To better understand the function of CsOPR3, the accumulation of the CsOPR3 protein under different treatments was investigated using specific monoclonal antibodies (mAbs) against CsOPR3. The CsOPR3 protein accumulated in the wounded leaves and reached its maximum level 3 h after treatment after which its expression decreased until the increase in its levels disappeared at 24 h (Fig. 1a). JA treatment improved the accumulation of CsOPR3, and the elevated level was maintained for 24 h (Fig. 1a). The results regarding CsOPR3 protein levels were consistent with the previously reported transcript expression of *CsOPR3*^23^. In addition, TLH treatment increased the transcript and protein levels of CsOPR3 from 3 to 24 h (Fig. 1a, b).

### CsOPR3 preferentially catalyzes the reduction of (+)-cis-OPDA

**Fig. 2:**
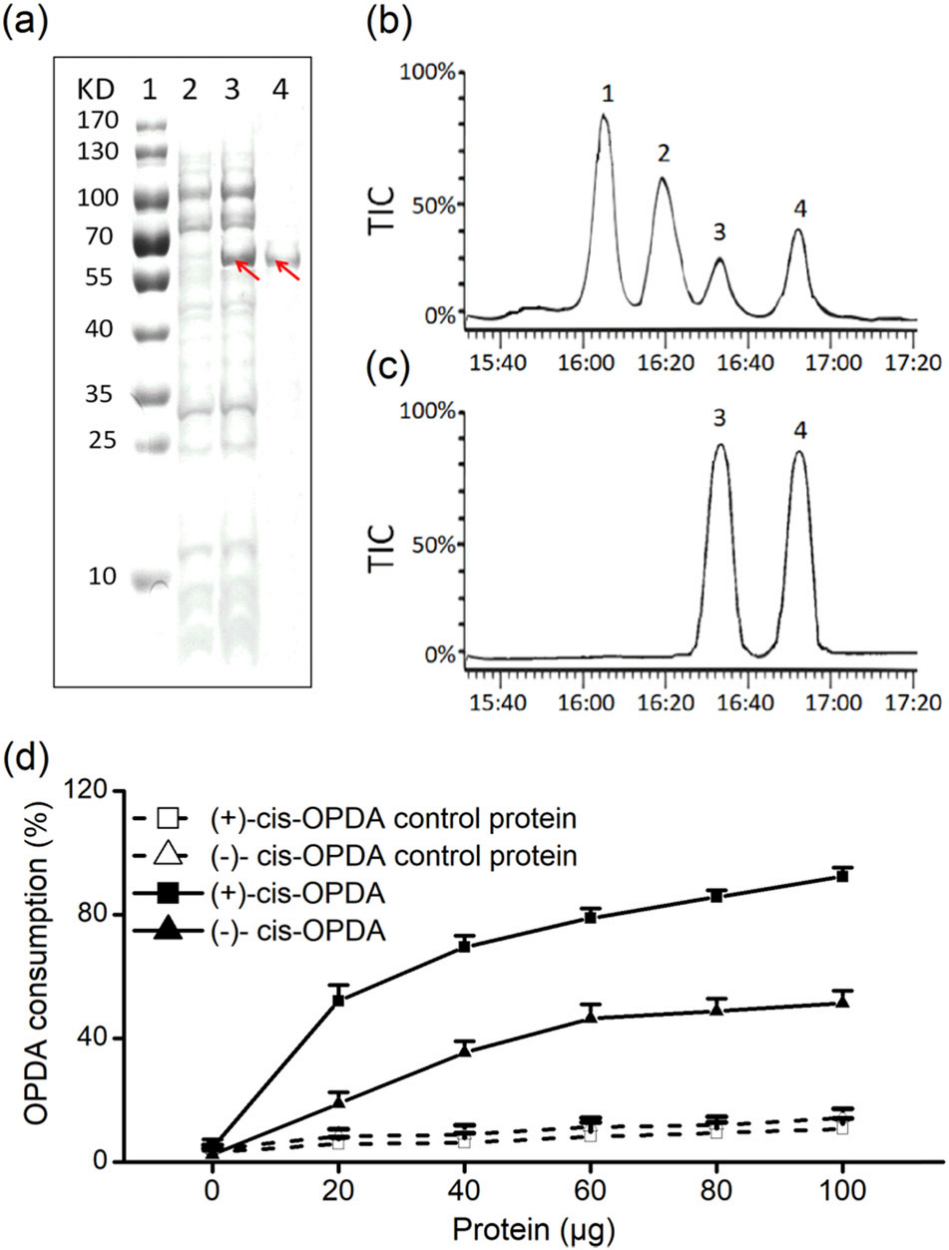
Analysis of the products of reactions catalyzed by the CsOPR3-His protein. **a** SDS-PAGE analysis of recombinant CsOPR3. The CsOPR3-His protein was expressed in *E. coli* and purified by ion-exchange chromatography. **1** molecular marker, **2** crude protein not induced by IPTG, **3** crude protein induced by IPTG, and **4** purified enzyme. **b** Total ion chromatograms of the products of the reactions of enantiomeric cis-OPDA with the CsOPR3-His protein. **c** Total ion chromatograms of products of the reaction of enantiomeric cis-OPDA with the control protein. peak **1**: (+)-cis-OPC-8:0; peak **2**: (−)-cis-OPC-8:0; peak **3**: (+)-cis-OPDA; peak **4**: (−)-cis-OPDA. **d** Relative OPDA consumption by the soluble protein fraction with or without the recombinant CsOPR3 protein. The reactions were carried out in 0.5 ml assay solution at 25 °C for 0.5 h with (+)-cis-OPDA or (−)-cis-OPDA as a substrate

To test the biological function of CsOPR3, the recombinant CsOPR3-His plasmid was transferred to and expressed in *Escherichia coli* BL21 (DE3) cells and the resultant expression was estimated by sodium dodecyl sulfate-polyacrylamide gel electrophoresis (SDS-PAGE). The recombinant protein encoded by CsOPR3 and part of pET32a (+) vector had a presumed molecular mass of 63.8 kDa. A specific band of the corresponding size was obtained through SDS-PAGE of the crude protein fraction from *E. coli* cells induced with isopropyl β-d-1-thiogalactopyranoside (IPTG) (Fig. 2a). This band was not found in the protein fraction from *E. coli* cells that were not induced by IPTG (Fig. 2a). The CsOPR3-His protein was used to evaluate the ability of CsOPR3 to catalyze the reduction of cis-OPDA to cis-OPC-8:0 by chiral capillary gas chromatography–mass spectrometry (GC–MS). The CsOPR3 protein can convert (+)-cis-OPDA to (+)-cis-OPC-8:0, the naturally occurring precursor of (+)-cis-JA (Fig. 2b). In addition, (−)-cis-OPDA can be reduced to (−)-cis-OPC-8:0 by the CsOPR3-His protein (Fig. 2b). No substrate conversion was detected in the presence of the control protein (Fig. 2c). Next, the enantiomeric preference of the CsOPR3-His protein was analyzed. The recombinant protein could reduce cis-OPDA and showed a strong preference for (+)-cis-OPDA. With (+)-cis-OPDA as a substrate, the crude protein fraction from *E. coli* cells containing CsOPR3 at concentrations of 20 and 100 μg in 0.2 ml assay solution consumed 52.2 and 92.3% of the substrate, respectively. With (−)-cis-OPDA as a substrate, the crude soluble protein fraction consumed 51.5% of the substrate at a concentration of 100 μg in 0.2 ml assay solution. This activity was markedly higher than that of the control protein (15.2%) (Fig. 2d). CsOPR3 exhibited OPR activity similar to that of AtOPR3^24^. These findings indicated that the CsOPR3 gene encoded a functional OPR, which is an excellent candidate for the biosynthesis of JA.

### Constitutive *CsOPR3* expression complements wound-induced JA production in the *opr3* mutant

**Fig. 3:**
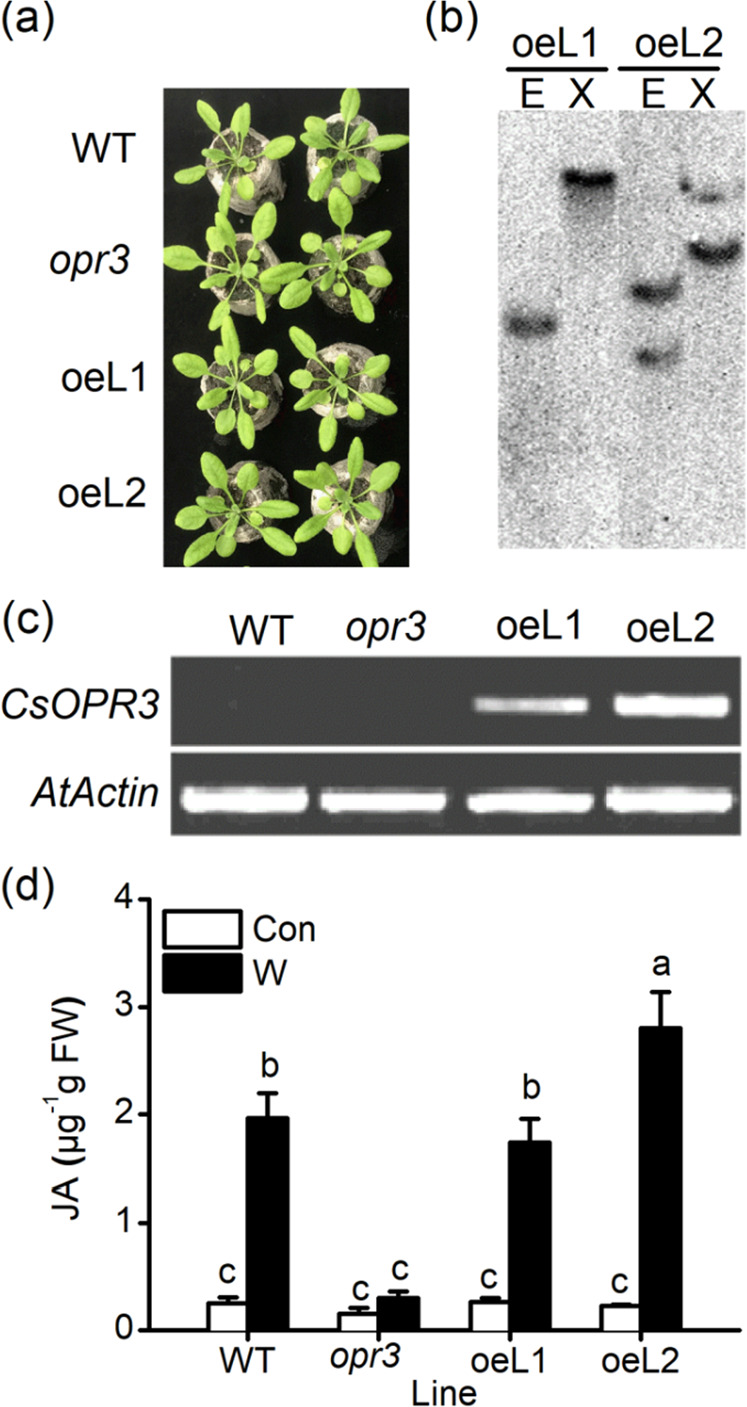
Complementary experimental analysis of wound-induced JA levels in the *Arabidopsis opr3* mutant following the overexpression of *CsOPR3*. **a** Growth phenotypes of WT, *opr3* and *opr3*-overexpressing *CsOPR3* (oeL1 and oeL2) lines in 20-day-old plants. **b** Identification of transgene copies in the two independent transgenic lines (oeL1 and oeL2). Total genomic DNA was extracted from transgenic *Arabidopsis* leaves and digested using *EcoR I* (E) or *Xba I* (X). (**c**) Confirmation of *CsOPR3* expression in *Arabidopsis* WT, *opr3*, oeL1, and oeL2 lines using RT-PCR. **d** Endogenous JA contents in *Arabidopsis* WT, *opr3*, oeL1, and oeL2 leaves (20 days old) with or without wounding treatment. The values are the means + SEs for five independent biological replicates. Letters indicate significant differences among JA levels in different *Arabidopsis* lines (*ANOVA*, *P* < 0.05).

To examine whether *CsOPR3* participates in JA synthesis, *CsOPR3* was overexpressed in the *Arabidopsis opr3* mutant, which shows a low JA level after wound treatment^25^. The *35S::CsOPR3* overexpression vector construct was inserted into *opr3*, and two T2 homozygous lines (oeL1 and oeL2) were obtained (Fig. 3a). Southern blot analysis showed that oeL1 harbored a single insertion, whereas oeL2 possessed two copies of the sequence (Fig. 3b). Figure 3c shows that the constitutive expression of *CsOPR3* was detected by real-time polymerase chain reaction (RT-PCR) in the two transgenic lines. Then, the JA level in leaves of wounded or non-wounded 3-week-old plants was measured. Figure 3d shows that wounding treatment increased JA levels in wild-type (WT) plants. Although the *opr3* plants produced little JA in response to wounding, the *opr3* mutant overexpressing *CsOPR3* showed the recovery of JA production after wounding (Fig. 3d). These results indicated that *CsOPR3* could function as the OPR for JA biosynthesis in tea plants. In oeL2, the wound-induced JA level was higher than that in the WT and oeL1 plants, which might be related to the two copies of *CsOPR3* in oeL2.

### CsOPR3 and JA are involved in the defense of tea plants against *C. fructicola*

**Fig. 4:**
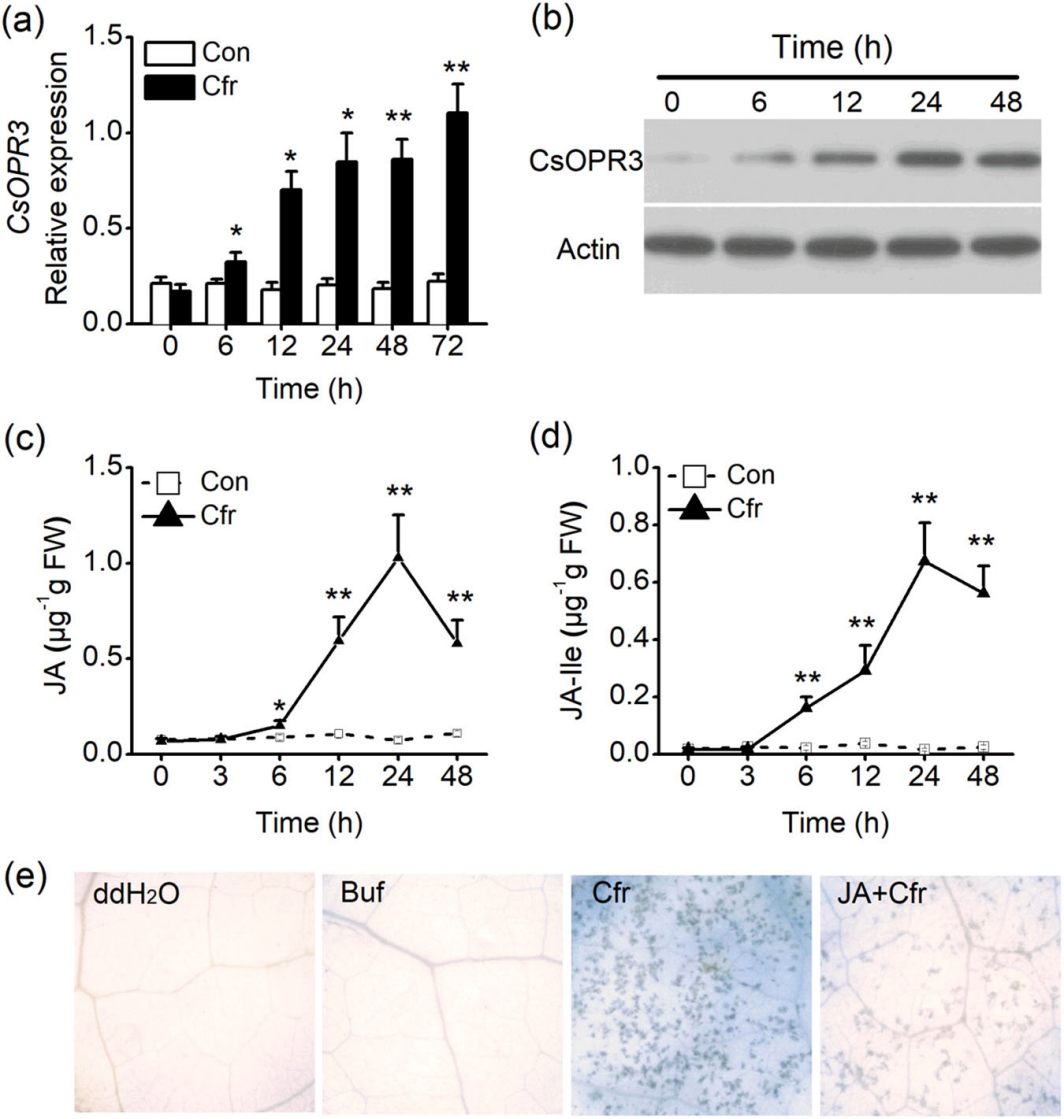
CsOPR3 and jasmonic acid (JA) are involved in the defense of tea plants against *C. fructicola*. **a**, **b** Transcript level and protein levels of CsOPR3 in tea leaves after *C. fructicola* treatment (Cfr). **c**, **d** Contents of JA (**c**) and JA-Ile (**d**) in tea leaves after *C. fructicola* treatment (Cfr) or the controls (Con). Values are presented as the means + SEs for five biological replicates. **e** Trypan blue staining for cell death in tea leaves at 6 days post inoculation. ddH_2_O, tea plants treated with ddH_2_O as the control for Cfr treatment; Buf, tea plants treated with 50 mM sodium phosphate buffer of pH 8 as the control for JA treatment; Cfr, tea plants infected with *C. fructicola* suspension at a concentration of 2 × 10^5^ spores per ml; JA + Cfr, tea plants treated with both JA and *C. fructicola*. Asterisks indicate significant differences between Cfr-treated and control plants (*t* test, *P < 0.05; **P < 0.01).

To investigate how *CsOPR3* responds to *C. fructicola* treatment, tea plants were inoculated with a *C. fructicola* spore suspension. The expression of *CsOPR3* increased at 6 h after treatment, and the increase was maintained for 24 h (Fig. 4a). Similar to the transcript results, *C. fructicola* treatment promoted the accumulation of the CsOPR3 protein (Fig. 4b). Consistent with the observed CsOPR3 expression, the contents of JA and JA-Ile began to accumulate 6 h after *C. fructicola* treatment, and reached a peak at 24 h (Fig. 4c, d). To test the effect of JA on the defense of tea plants against *C. fructicola*, tea plants were pretreated with JA and infected with *C. fructicola. C. fructicola* induced serious cell death six days after treatment. The disease symptoms induced by *C. fructicola* were suppressed by JA application. No staining was detected in leaves treated with ddH_2_O (the control for *C. fructicola* treatment) or the sodium phosphate buffer (SPB) (the control for JA treatment) (Fig. 4e). These data indicated that CsOPR3 and JA were involved in the defense of tea plants against *C. fructicola*.

### Generation of *OPR3p::GUS* transgenic *Arabidopsis* lines

**Fig. 5:**
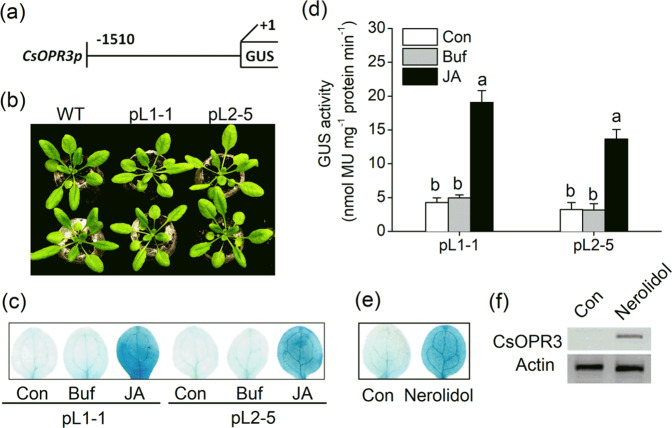
GUS activities in O*PR3p::GUS* transgenic *Arabidopsis* plants. **a** Diagram of the *OPR3p::GUS* fusion vector. The number indicates the *CsOPR3* 5’ promoter end point relative to the transcriptional start site. **b** Growth phenotypes of WT plants and *OPR3p::GUS* lines (pL1-1 and pL2-5) at 20 days of age. **c**, **d** GUS staining (**c**) and GUS activity (**d**) in 15-day-old *OPR3p::GUS Arabidopsis* seedlings, 12 h after treatment with jasmonic acid (JA), buffer (Buf), or the controls (Con). Values are presented as the means + SEs for five biological replicates. **e** GUS staining of 15-day-old *OPR3p::GUS Arabidopsis* seedlings 2 h after exposure to (*E*)-nerolidol or the controls (Con). **f** Western blot analysis of the accumulation of CsOPR3 in tea leaves exposed to (*E*)-nerolidol or the controls (Con). Letters indicate significant differences among treatments (ANOVA, *P* < 0.05).

A 1.51-kb sequence of the promoter region was cloned and analyzed by using PlantCARE software (Fig. S1). The promoter sequence contained a typical core promoter element (TATA-box), an enhancer element (CAAT-box), and a transcriptional start site. We focused on *cis*-elements associated with defense reactions and found several *cis*-acting elements related to plant responses to phytohormones (DOFCOREZM and ERELEE4), wounding (W-box), and light (GT1-motif), and other defense-responsive elements such as the BIHD1-motif, GT-1-motif, and WRKY71OS^26^ (Table S2). The promoter was fused to a GUS gene (Fig. 5a), and two independent lines in *Arabidopsis* (*OPR3p::GUS* lines, pL1-1 and pL2-5) were finally generated. No apparent growth difference was observed between WT plants and the transgenic lines (Fig. 5b). To determine whether the pL1-1 and pL2-5 lines respond to stress, we performed JA treatment. Three hours after JA treatment, the foliar tissues of pL1-1 and pL2-5 leaves were stained blue by GUS staining. Minimal staining was detectable in control and buffer-treated leaves (Fig. 5c). Quantitative analysis of GUS activity showed that JA treatments remarkably increased GUS activity in transgenic *Arabidopsis* leaves (Fig. 5d). The detectable staining and increased GUS activity were consistent with the results regarding CsOPR3 protein accumulation in JA-treated tea plants (Fig. 1a). Thus, GUS activity in transgenic *Arabidopsis* represented the change in CsOPR3 in tea plants.

### (*E*)-Nerolidol was screened as a candidate volatile signal

As important signal compounds released by plants, VOCs include various chemicals that constitute a large natural compound library. An important manifestation of the VOC-induced activation of plant defense mechanism is the induction of a JA burst. Thus, the *OPR3p::GUS* lines are good bioreactors for rapid screening. To screen volatile signals from VOCs, 15-day-old seedlings of the pL1-1 or pL2-5 line were exposed to different compounds for 0.5 h, and GUS staining was performed. The results showed that (*E*)-nerolidol, a terpenoid volatile, increased GUS activity in the leaves of *OPR3p::GUS* lines. The (*E*)-nerolidol-treated *Arabidopsis* leaves were stained clear blue by GUS staining (Fig. 5e). In the tea plants, the CsOPR3 protein also accumulated after (*E*)-nerolidol treatment (Fig. 5f).

### Effect of (*E*)-nerolidol on signaling events in tea plants

**Fig. 6:**
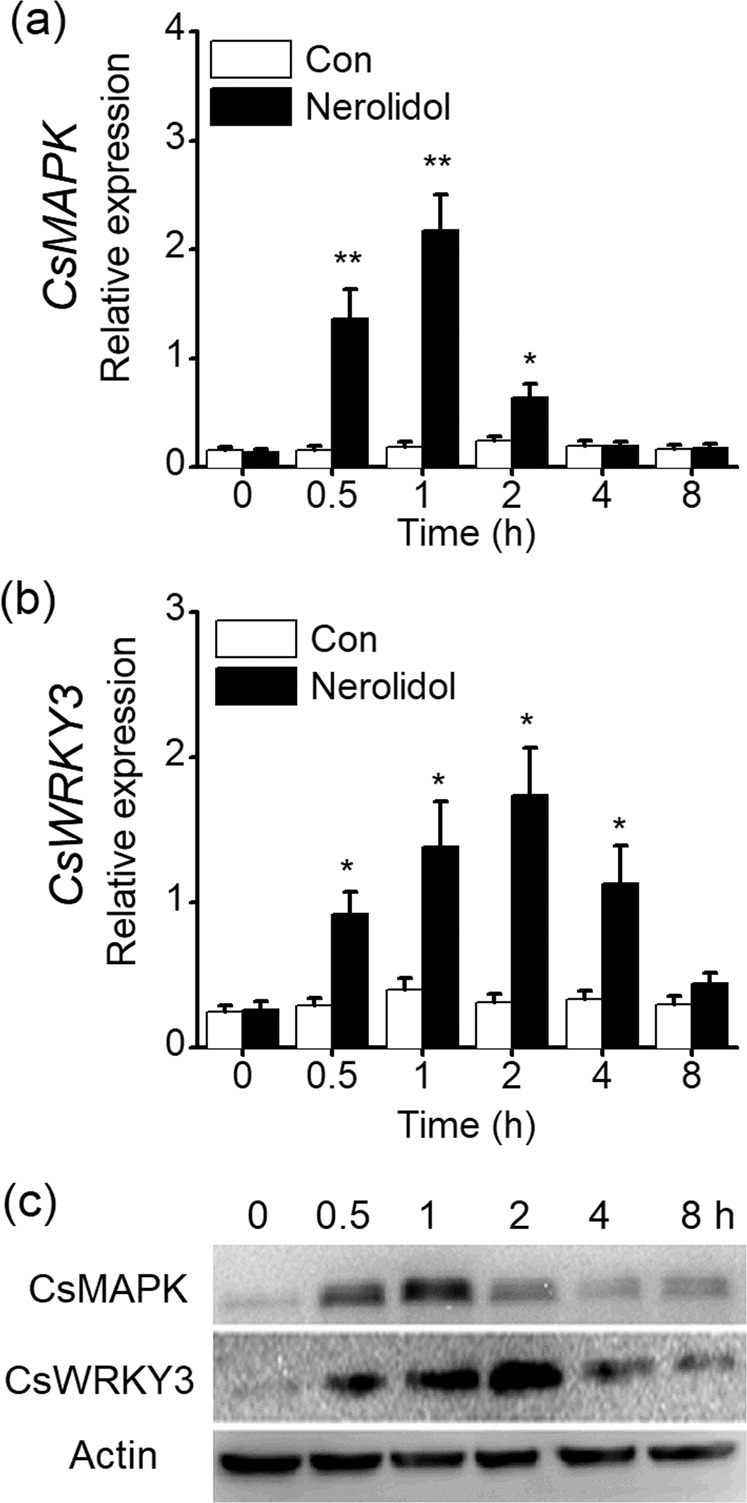
(*E*)-Nerolidol regulates the mitogen-activated protein kinase (MAPK) and WRKY genes in tea plants. **a**, **b** Transcript levels of *CsMAPK* (**a**) and *CsWRKY3* (**b**) in tea leaves exposed to (*E*)-nerolidol and the controls (Con). Values are presented as the means + SEs for five biological replicates. **c** Western blot analysis of the protein accumulation of CsMAPK and CsWRKY3 in tea leaves exposed to (*E*)-nerolidol. Asterisks indicate significant differences between tea leaves treated with (*E*)-nerolidol and the controls (*t* test, *P < 0.05; **P < 0.01).

**Fig. 7:**
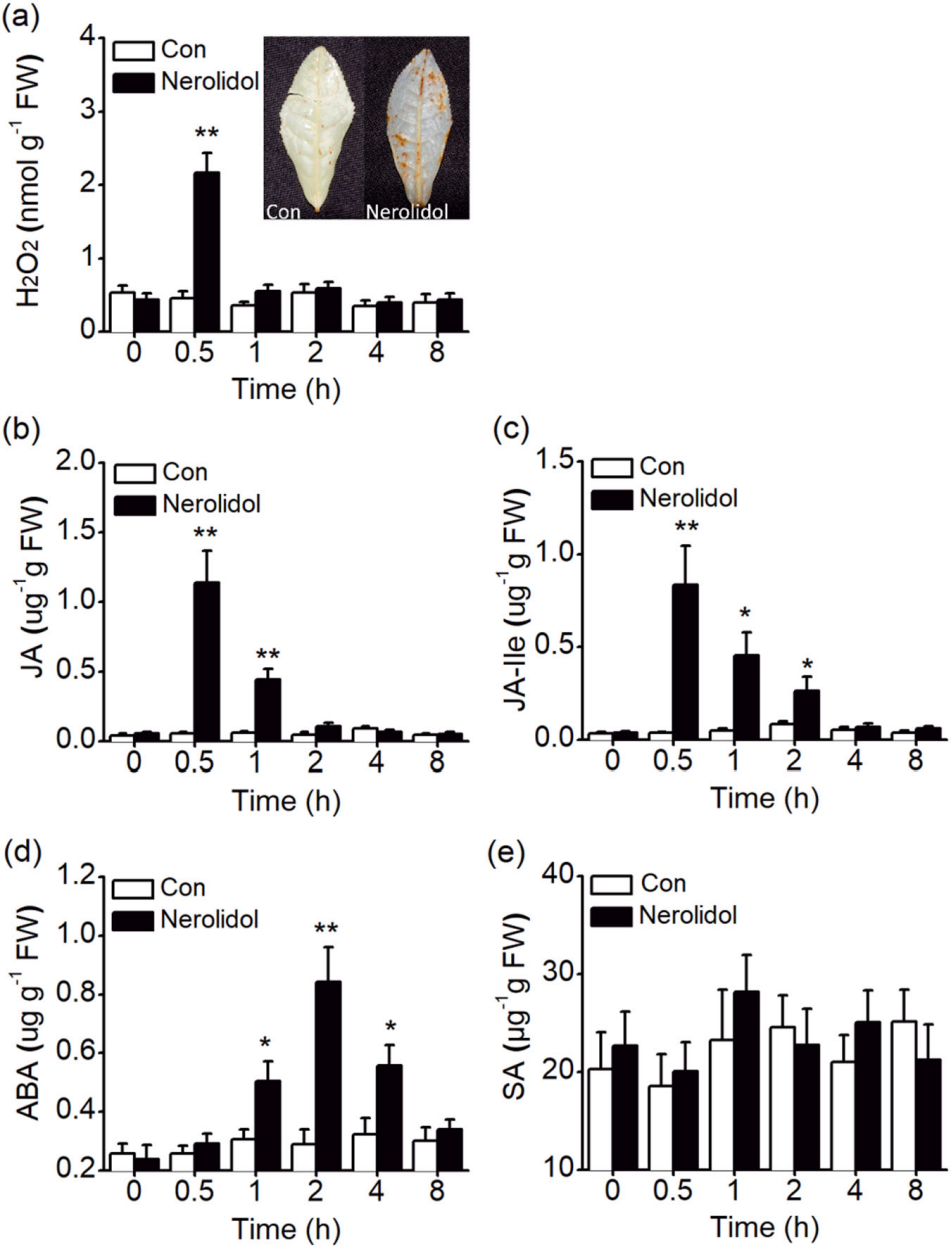
Effect of (*E*)-nerolidol on the levels of signaling molecules related to defense in tea plants. **a** H_2_O_2_ contents in control (Con) and (*E*)-nerolidol-treated plants. The inset shows the DAB staining of H_2_O_2_ levels in tea leaves exposed to (*E*)-nerolidol and the controls (Con). **b**–**e** Contents of JA (**b**), JA-Ile (**c**), ABA (**d**), and SA (**e**) in control (Con) and (*E*)-nerolidol-treated plants. Values are presented as the means + SEs for five biological replicates. Asterisks indicate the significant differences between (*E*)-nerolidol-treated plants and controls (*t* test, *P < 0.05; **P < 0.01).

The early events of plant defense responses are often regulated by MAPK and WRKY genes^27,28^. Thus, we investigated whether treatment with (*E*)-nerolidol altered the expression of *CsMAPK* and *CsWRKY3* in tea plants. The transcript level of *CsMAPK* was increased at 0.5 h and reached a peak at 1 h after treatment. Treatment with (*E*)-nerolidol also increased the transcript level of *CsWRKY3* at 0.5 h and peaked at 2 h (Fig. 6a, b). Consistent with the gene expression results, (*E*)-nerolidol treatment increased the protein accumulation of CsMAPK and CsWRKY3 (Fig. 6c). In addition, the levels of four signaling molecules, H_2_O_2_, JA, JA-Ile, and ABA were increased by (*E*)-nerolidol. The levels of H_2_O_2_, JA, and the JA derivative JA-Ile increased rapidly and peaked at 0.5 h but decreased rapidly at 1–2 h (Fig. 7a–c). Thirty minutes after (*E*)-nerolidol treatment, the foliar tissues of tea turned brown upon 3,3-diaminobenzidine (DAB) staining. Little staining was detected in the control leaves (inset of Fig. 7a). ABA levels increased in (*E*)-nerolidol-treated tea leaves at 1 h and peaked at 2 h (Fig. 7d). However, (*E*)-nerolidol did not affect the SA contents in tea leaves (Fig. 7e).

### (*E*)-Nerolidol activates resistance against TLH

**Fig. 8:**
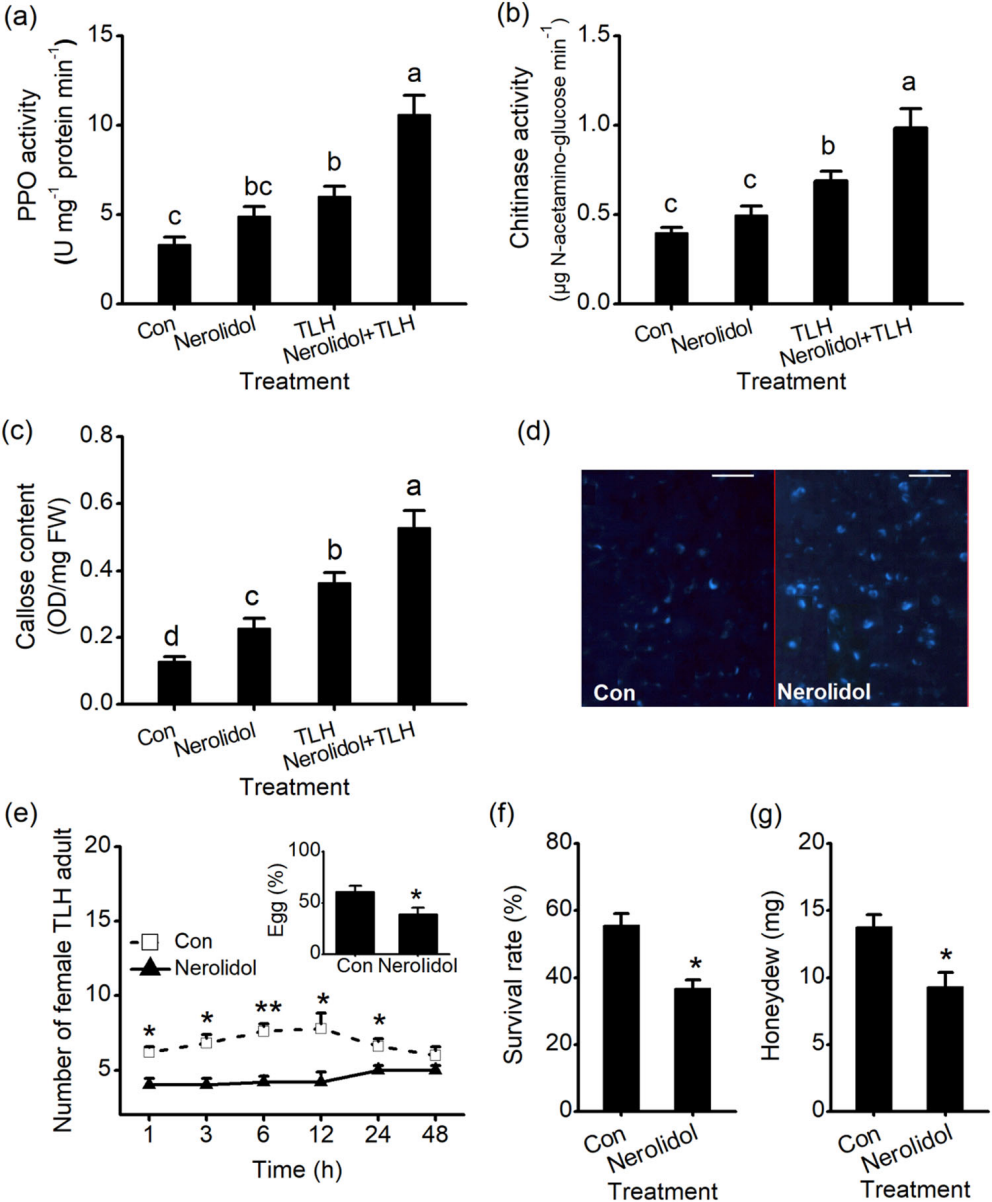
Effect of (*E*)-nerolidol on the defense of tea plants against *E. onukii*. **a**–**c** PPO activities (**a**), chitinase activities (**b**), and callose contents (**c**) in tea plants under Con, Nerolidol, TLH, and Nerolidol + TLH treatments. For the Nerolidol treatment, tea plants were exposed to (*E*)-nerolidol for 0.5 h and ventilated for 0.5 h, and the second leaves were collected for measurement 12 h after treatment. For the TLH treatment, tea plants were infested with15 *E. onukii* for 12 h. For the Nerolidol + TLH treatment, tea plants were exposed to (*E*)-nerolidol for 0.5 h, ventilated for 0.5 h, and infested with *E. onukii* for 12 h. Values are presented as the means + SEs for five biological replicates. **d** Callose deposition in control leaves (Con) and (*E*)-nerolidol-treated leaves. Aniline blue was used to stain tea leaves to detect callose. Scale bars represent 50 μm. **e** The numbers of *E. onukii* female adults on (*E*)-nerolidol-treated plants and controls. The inset shows the percentage of TLH eggs on pairs of plants, 3 days after TLH was released. Values are presented as the means + SEs for six independent biological replicates. **f** The survival rates of TLH nymphs fed on (*E*)-nerolidol-treated plants and control plants 3 days after the nymphs were placed on the plants. Values are the means + SEs for six biological replicates. **g** The amount of honeydew per day per TLH female adult fed on (*E*)-nerolidol-treated plants or control plants. Values are the means + SEs for fifteen biological replicates. Letters represent significant differences among the four treatments (ANOVA, *P* < 0.05). Asterisks indicate the significant differences between the treatments and controls (*t* test, *P < 0.05; **P < 0.01).

PPO and chitinase activities in (*E*)-nerolidol-treated leaves were 1.48-fold and 1.24-fold higher than those in the control, respectively. (*E*)-Nerolidol treatment also increased the TLH-elicited activity of PPO and chitinase. For example, PPO levels in plants treated with (*E*)-nerolidol and TLH were 1.77-fold higher than those in TLH-infested leaves (Fig. 8a, b). (*E*)-Nerolidol also increased the basal and TLH-induced callose contents. The callose contents of (*E*)-nerolidol-treated leaves were 1.76-fold higher than those in the control. TLH infestation induced higher callose contents in (*E*)-nerolidol-treated plants than in TLH-infested plants without (*E*)-nerolidol treatment (Fig. 8c). Consistent with this result, the (*E*)-nerolidol-treated leaves exhibited high callose deposition (Fig. 8d). We subsequently examined the effects of (*E*)-nerolidol on the defense of tea plants against TLH. When tea plants were exposed to female TLH colonies under different treatments, TLH was more often observed on the control plants than on the plants exposed to (*E*)-nerolidol (Fig. 8e). Similarly, TLH female adults deposited considerably fewer eggs on (*E*)-nerolidol-treated plants than on control plants (inset of Fig. 8e). Moreover, the survival rates of TLH nymphs that fed on (*E*)-nerolidol-treated plants were only 66.2% of those that fed on control plants (Fig. 8f). TLH female adults that fed on control plants secreted large amounts of honeydew, and honeydew secretion was reduced by 42.9% on plants treated with (*E*)-nerolidol (Fig. 8g), indicating that (*E*)-nerolidol treatment negatively affected the amount of food intake of TLH.

### Exogenous (*E*)-nerolidol reduces the susceptibility of tea plants to *C. fructicola*

**Fig. 9:**
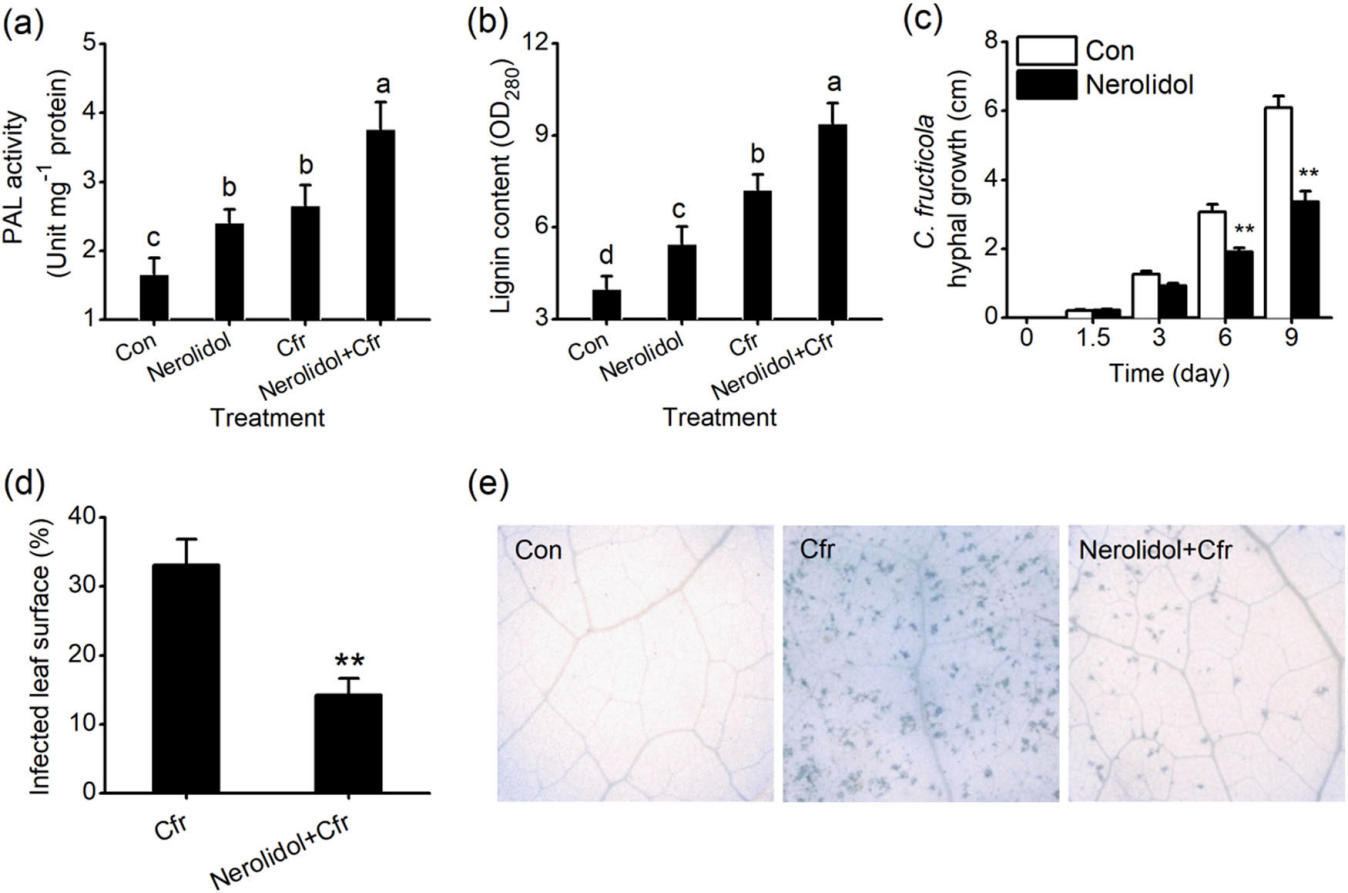
Effect of (*E*)-nerolidol on the susceptibility of tea plants to *C. fructicola*. **a**, **b** PAL activity (**a**) and lignin content (**b**) in tea plants under different treatments. Con, control; Nerolidol, tea plants were exposed to (*E*)-nerolidol for 0.5 h, ventilated for 0.5 h, and collected for measurement 24 h after treatment; Cfr, tea plants were infected with *C. fructicola* for 24 h; Nerolidol + Cfr, tea plants were exposed to (*E*)-nerolidol for 0.5 h, ventilated for 0.5 h, and infected with *C. fructicola* for 24 h. **c** Hyphal growth of *C. fructicola* under (*E*)-nerolidol treatment compared with the controls (Con) at different time points. **d** The infected surface areas of leaves at 9 d after *C. fructicola* treatment (Cfr) or *C. fructicola* and (*E*)-nerolidol treatment (Nerolidol + Cfr). Values are the means + SEs for nineteen biological replicates. **e** Trypan blue staining for cell death on tea leaves at 6 d after different treatments. Con, tea plants were treated with ddH2O as controls; Cfr, tea plants were infected with a *C. fructicola* suspension with a concentration of 2 × 10^5^ spores per ml; Nerolidol + Cfr, tea plants were treated with (*E*)-nerolidol and *C. fructicola*. Letters indicate significant differences among treatments (ANOVA, *P* < 0.05). Asterisks indicate the significant differences between two different treatments (*t* test, *P < 0.05; **P < 0.01).

PAL activity increased in tea leaves treated with (*E*)-nerolidol or *C. fructicola*. PAL activities in the leaves of (*E*)-nerolidol-treated plants or *C. fructicola*-treated plants were 1.45-fold or 1.60-fold higher, respectively, than those in the controls (Fig. 9a). In addition, *C. fructicola*-elicited PAL activity was increased in plants treated with (*E*)-nerolidol (Fig. 9a). Moreover, (*E*)-nerolidol had a positive effect on lignin accumulation in tea plants. (*E*)-Nerolidol treatment caused a significant 1.37-fold increase in lignin content. Furthermore, treatment with (*E*)-nerolidol increased the lignin content in *C. fructicola*-infested plants. Lignin contents in plants treated with (*E*)-nerolidol and *C. fructicola* were 1.30-fold higher than those in *C. fructicola*-infested plants without (*E*)-nerolidol treatment (Fig. 9b). Thus, we investigated whether treatment with (*E*)-nerolidol altered the susceptibility of tea plants to *C. fructicola*. We first determined the effect of (*E*)-nerolidol on the growth of *C. fructicola* and observed disease symptoms using trypan blue staining for the detection of cell death. (*E*)-Nerolidol inhibited fungal growth in vitro. Six days after treatment, the length of the fungal hyphae on (*E*)-nerolidol-treated plants was only 60.2% of that on the control plants (Fig. 9c). The *C. fructicola*-treated tea leaves showed clear and typical disease symptoms of anthracnose nine days after treatment. As expected, (*E*)-nerolidol reduced the symptoms induced by *C. fructicola*. The average infected surface area of leaves treated with (*E*)-nerolidol followed by *C. fructicola* infection was 43.1% smaller than that in *C. fructicola*-infected plants (Fig. 9d). Trypan blue staining showed that (*E*)-nerolidol suppressed *C. fructicola*-induced cell death in tea leaves (Fig. 9e).

## Discussion

### *CsOPR3* as a marker gene for the rapid screening of defense elicitors

Fusions of the promoter regions of marker genes with the GUS reporter gene (*Promoter::GUS*) can be used to determine the function of the marker genes using forward chemical genetics. After induction treatment, the promoter drives the production of the GUS protein in plant tissues. The GUS protein can react with the substrate X-gluc to generate visible blue coloration. The color is stable, and the resultant specimen can be stored for several days. To date, various studies have used this blue coloration as a trait for the screening of plant disease resistance elicitors^29,30^. JA signaling can be used as a marker pathway for the identification of synthetic chemical elicitors for the biological control of pests and diseases because of its crucial role in the regulation of plant defense^31,32^. *CsOPR3* is a putative OPR gene responsible for JA biosynthesis. This gene shows sensitive responses to wounding and herbivore infestation and is suitable as a candidate marker gene^23^. We demonstrated that *CsOPR3* encodes a JA biosynthesis enzyme and established a rapid screening system in the present study.

Refining the assessment of expression levels from the transcript level to the protein level can improve the reliability of the experimental data. Western blot analysis showed that mechanical wounding and JA treatment, which cause plants to produce defense reactions similar to those induced by herbivore attacks^33^, increased the accumulation of the CsOPR3 protein in tea leaves (Fig. 1a). As TLH feeds via cell rupture^34^, its feeding caused lasting CsOPR3 expression (Fig. 1), which was related to the continuous damage resulting from TLH feeding and the herbivore-specific elicitors (e.g., β-glucosidase and volicitin) derived from the regurgitant^35^. Next, we demonstrated that CsOPR3 functioned as a reductase specific for the natural (+)-cis-OPDA enantiomer (Fig. 2). Therefore, similar to AtOPR3 and SlOPR3, CsOPR3 functions to reduce the natural JA precursor (+)-cis-OPDA to (+)-cis-OPC-8:0. Thus, CsOPR3 is a good candidate enzyme for catalyzing the biosynthesis of JA in plants. As expected, *CsOPR3* overexpression in the *opr3* mutant recovered the capability to produce JA after wounding (Fig. 3).

CsOPR3 responded to pathogen infection, and its transcription and protein levels increased after *C. fructicola* infection. JA and JA-Ile levels were associated with the increase in the *CsOPR3* transcript during pathogen infection (Fig. 4). The correlation between CsOPR3 expression and JA content was consistent with the function of OPR3 in octadecanoid signaling in other plants^24^. The response of OPR to the pathogens is also found in wheat. In wheat, *TaOPR2* is upregulated by the leaf rust pathogen *Puccinia striiformis* f. *sp. tritici* and stripe rust pathogen *Puccinia recondite* f*. sp. tritici*^36^. In addition, our results showed that exogenous JA treatment enhanced the defense of tea plants against *C. fructicola* (Fig. 4). This result was similar to the findings of Li et al.^37^ who showed that MeJA application reduced the susceptibility of tea plants to *Colletotrichum gloeosporioides*, another causal agent of anthracnose disease. Collectively, the results indicate that CsOPR3-mediated JA signaling is involved in the defense of tea plants against *C. fructicola*.

To establish *promoter::GUS* lines, we cloned the promoter of *CsOPR3*. Several putative cis-regulatory elements that respond to plant hormones (DOFCOREZM, ERELEE4), wounding (W-box), and light (GT1-motif) and other defense-regulatory elements (BIHD1-motif, GT1-motif, and WRKY71OS)^26^ were found in the 1.51-kb promoter sequence of *CsOPR3* (Table S2). When the *OPR3p::GUS* construct was transformed into *Arabidopsis*, the transgenic lines displayed visible blue color, which was consistent with the protein and transcript expression levels in tea leaves after JA treatment (Fig. 5), indicating that *OPR3p::GUS* lines could be used as a rapid system for the screening of synthetic chemical elicitors. Moreover, the discovery of the candidate chemical elicitor (*E*)-nerolidol confirmed that this system is reliable and robust.

### (*E*)-Nerolidol as a volatile signal of tea defense

(*E*)-Nerolidol is a major terpenoid volatile and is specifically released in response to chewing insect infestation or continuous mechanical damage^38^. Similar to other VOCs, (*E*)-nerolidol can influence the behavior of parasitic wasps or predators, thereby preserving plant safety. For example, the attraction of the predatory mite *Phytoseiulus persimilis* to (*E*)-nerolidol has been well demonstrated^39^. However, the role of (*E*)-nerolidol in inducing defense against invaders has been less studied. In the present study, we found that the foliar tissues of *OPR3p::GUS* lines treated with (*E*)-nerolidol were stained dark blue upon X-gluc staining using the rapid screening system (Fig. 5e). As expected, the accumulation of the CsOPR3 protein in tea leaves was increased by (*E*)-nerolidol treatment (Fig. 5f). Chemical and molecular analyses showed that the exogenous application of (*E*)-nerolidol activated the MAPK cascades and WRKY (Fig. 6) and increased the levels of JA, ABA, H_2_O_2_ (Fig. 7), and defense chemicals, such as PPOs, PAL, chitinase, callose, and lignin (Figs. 8, 9), which have extensive natural anti-herbivore or anti-pathogen effects. Finally, the (*E*)-nerolidol treatment of tea plants directly negatively affected the feeding, fecundity, and survival rate of TLH (Fig. 8e–g). Moreover, (*E*)-nerolidol decreased the disease symptoms and leaf cell death caused by *C. fructicola* infection in tea plants (Fig. 9c–e). This study is the first to show that (*E*)-nerolidol functions as a volatile signal and provides robust defense against a piercing herbivore and a pathogen in tea plants.

MAPKs and WRKYs play vital roles in the regulation of defense, stress, and development. They respond rapidly to herbivore/pathogen-related stress or stimuli, leading to the activation of specific downstream targets to elicit the biosynthesis of defense-related signal molecules through synergistic or antagonistic interactions^27,28^. The rapid induction of CsMAPK and CsWRKY3 by (*E*)-nerolidol in tea plants (Fig. 6) suggested that both genes play significant roles in the perception of and response to VOCs. In many plants, MAPK and WRKY genes take part in the perception of diverse chemical elicitors. For example, cryptogein and oligogalacturonides elicit the expression of salicylic acid-induced protein kinase and wound-induced protein kinase in tobacco^40,41^. In rice, the expression of MAPK and WRKY genes can be increased by 2,4-D^12^. Recently, we found that tea plants treated with the disease resistance elicitor laminarin showed the rapid accumulation of CsMAPK and CsWRKY3, with high levels of SA but not JA^13^. The opposite situation was found in the present study: the (*E*)-nerolidol-induced increases in CsMAPK and CsWRKY3 caused higher levels of JA but not SA, suggesting that their function in regulating JA or SA biosynthesis might differ from that of defense elicitors. We speculate that when MAPK/WRKY-mediated early events are activated by laminarin or (*E*)-nerolidol, these elicitors may show differences in the regulation of several modules related to the crosstalk between SA and JA, such as non-expressor of pathogenesis-related genes1 (NPR1). Then, specific plant defense responses are ultimately fine-tuned via the mediation of different pathways^42^. These hypotheses must be investigated further.

The exposure of tea leaves to (*E*)-nerolidol increased H_2_O_2_, JA, and ABA production (Fig. 7). These signaling molecules occur ubiquitously and exhibit distinct but overlapping patterns in modulating the growth, development, and defense response of plants^8,43^. As a reduced and chemically reactive molecule among the ROS, H_2_O_2_ is often detected under pathogen infection and stimulation with chemicals such as JA^2,44,45^. H_2_O_2_ can activate defense genes, most of which are involved in responses to oxidative stress and induce a hypersensitive response^46^. In addition, H_2_O_2_-mediated cell wall modifications play vital roles in plant defense against pathogens and piercing–sucking insects^47^. The present study identified H_2_O_2_ accumulation as the earliest detectable cytological response of (*E*)-nerolidol-treated tea leaves (Fig. 7a). As major regulators of defense responses, JA and JA-Ile exert a substantial effect on herbivore and pathogen performance^9^. Furthermore, the JA burst is an important mechanism whereby VOCs induce plant resistance. VOCs can cause plants to enter a “priming” state, resulting in a stronger and faster response to subsequent insect herbivory. The “priming” effect is closely related to JA signaling^19,21^. Although our studies showed the induction of increased JA and JA-Ile levels (Fig. 7b, c) and defense responses in herbivore/pathogen-infested tea plants by (*E*)-nerolidol (Figs. 8, 9), the possible “priming” effect of (*E*)-nerolidol on tea plants requires further investigation. The production of ABA and callose in tea plants was also increased by (*E*)-nerolidol (Figs. 7d and 8c, d). ABA can increase the antioxidant capacity when plants suffer stress^48^. An important function of ABA is the induction of the synthesis of callose, which can contribute to resistance to pathogen penetration^49^. Such callose sealing also prevents sucking herbivores from ingesting sap at sites where the stylet is inserted into the plants^43,50^. Thus, the increased ABA and callose deposition induced by (*E*)-nerolidol contribute to tea resistance against TLH and *C. fructicola*.

In addition to callose, (*E*)-nerolidol triggered high production of chitinase, PPO, PAL, and lignin. The TLH- or pathogen-elicited levels of these defense-related compounds were also increased by (*E*)-nerolidol treatment (Figs. 8, 9). The cell walls of insects/fungi can be hydrolyzed by chitinase^51^. The chitin fragments produced in this process can also amplify plant defense^52^. Large amounts of PPOs exist in tea plants. PPOs can directly reduce the food intake, fecundity, and survival rate of herbivores^53^. Thus, the increased accumulation of chitinase and PPO may partly explain the higher resistance of (*E*)-nerolidol-treated tea plants to *C. fructicola* and TLH. (*E*)-Nerolidol positively regulates the biosynthesis of lignin (Fig. 9b), which can increase the hardness of plant tissues and act as the first layer of the physical barrier, thereby preventing invaders from penetrating leaf tissue^54^. The increased lignin contents agree with the enhancement activity of PAL (Fig. 9a), which functions in the biosynthesis of lignin and plays a crucial role in defense against small brown planthoppers and fungal elicitors^55,56^. Therefore, high lignin and PAL levels may also contribute to the resistance of (*E*)-nerolidol-treated tea plants to TLH and *C. fructicola*.

In summary, to determine the defense signals of tea plants involving VOCs, we identified *CsOPR3* as a marker gene and set up a rapid screening method based on the *OPR3p::GUS* reporter in *Arabidopsis*. Using the transgenic lines, we identified (*E*)-nerolidol as a volatile signal involved in the defense of tea plants against TLH and *C. fructicola*. These data indicated that the activation of plant defenses using volatile signals is probably a valuable alternative strategy for preventing herbivore infection and restricting pathogen spreading.

## Materials and methods

### Plant, insects, and fungi

Three-year-old tea (*C. sinensis* L.) seedlings of “Longjing 43” were selected for the present study. Plants were grown in a controlled growth chamber at 26 ± 2 °C under a 12 h photophase, and 80% relative humidity (RH) at the Tea Research Institute of the Chinese Academy of Agricultural Sciences (TRICAAS, 30 °10′N, 120 °5′E), Hangzhou, China. The seeds of *Arabidopsis* Columbia WT and *opr3* mutant plants were ordered from the platform of the Arabidopsis Biological Resource Center. *Arabidopsis* lines were grown in soil in an illuminated incubator (22 °C, 16 h light: 8 h dark). TLH nymphs were obtained from tea fields of TRICAAS and fed fresh “Longjing 43” shoots in enclosed net cages in a glasshouse (26 ± 2 °C, 12 h photophase, 80% RH). The fungal pathogen *C. fructicola* was kindly supplied by Dr. Li Xin of TRICAAS. The fungus was identified by PCR with primers based on the sequences of the Apn2-Mat1-2 intergenic spacer and glutamine synthetase as described by Wang et al.^16^ and Weir et al.^57^.

### Plant treatments

#### TLH treatment

Each tea plant was infested with 15 TLH adults that were starved for 6 h. A protective mesh sleeve was used to prevent insect escape from the treated leaves.

#### Wound treatment

For wound treatments (W), a needle patch containing 20 pricks was used to damage the second leaf of tea plants. Each treated leaf was punctured with the needle patch ten times. The leaves of *Arabidopsis* plants were wounded using a needle patch with ten pricks. Each treated leaf was punctured with the needle patch five times. Untreated tea or *Arabidopsis* plants were chosen as controls (Con).

#### JA treatment

Each tea plant was separately sprayed with 10 ml of freshly prepared JA (150 μg ml^−1^) in 50 mM SPB at pH 8.

#### *C. fructicola* treatment

The foliar portion of tea plants was inoculated by spraying with 10 ml of a *C. fructicola* suspension at a concentration of 2 × 10^5^ spores per milliliter.

#### (E)-Nerolidol treatment

A silicon rubber septum was used as the odor dispenser. (*E*)-Nerolidol (10 μl) was added to the septum and ventilation was performed for 5 h, followed by storage at −20 °C. The septa were individually suspended in a sealed acrylic odor container with a diameter of 25 cm and a height of 65 cm to preliminarily treat the tea plants for 0.5 h. Within this time, the chemical was released at rates of 320–600 ng h^−1^, to approximate the release rates of tea plants^58^. The container was transferred to a ventilated place for 0.5 h before the experiments. The transgenic *Arabidopsis* seedlings were treated by suspending cotton wool with 5 μl of (*E*)-nerolidol in a sealed acrylic odor container with a diameter of 5 cm and a height of 10 cm for 0.5 h.

### Enzyme assay for CsOPR3

The enzyme activity assay of CsOPR3 was carried out according to the method of Tani et al.^57^, with 5 μg of purified CsOPR3-His recombinant protein and 7.5 μg of (+)-cis-OPDA or (−)-cis-OPDA as the substrate. Chiral GC–MS (Agilent, USA) was conducted to identify the reaction product and examine the enantiomer preference of CsOPR3. The reaction products were identified and quantified as described by Tani et al.^59^.

### Immunoblot analyses

CsOPR3-, CsMAPK-, and CsWRKY3-specific mAbs were produced by HuaAn Bioscience Technology (Hangzhou, China). The methods used for the production and purification of the mAbs were as described by Zhang et al.^60^. The total protein of tea leaves was isolated using the plant total protein extraction kit (Sigma-Aldrich) according to the manufacturer’s instructions. The protein samples were isolated by SDS-PAGE and transferred to polyvinylidene difluoride membranes for immunoblot assays.

### RNA preparation and transcript analysis

The total RNA of leaf samples was extracted with the Total RNA Isolation System (Invitrogen, CA, USA) following the manufacturer’s instructions. Reverse transcription was carried out using Superscript II (Invitrogen) Reverse Transcriptase. The quantitative RT-PCR (qRT-PCR) assay was carried out in a CFX96^™^ real-time system (Bio-Rad) with the Premix ExTaq Kit (TaKaRa). The qRT-PCR conditions consisted of a preliminary step at 95 °C for 2 min, followed by 40 cycles of denaturation at 95 °C for 10 s, annealing and extension step at 58 °C for 30 s. The tea GAPDH gene (GenBank no.: GE651107) was used to normalize transcript abundance. The primers used for qRT-PCR are listed in Supplementary Table S1.

### Generation of transgenic *Arabidopsis* lines

Total genomic DNA was extracted from fresh tea leaves using a plant genomic DNA kit (Tiangen, China). The 1.51-kb promoter region of *CsOPR3* (Fig. S1) was amplified by PCR using primer pairs (Table S1) designed according to the sequences supported by the Tea Plant Information Archive (http://tpia.teaplant.org/; Locus Name: TEA029800.1). PlantCARE software (http://bioinformatics.psb.ugent.be/webtools/plantcare/html/) was used to identify regulatory motifs in the promoter. Transcription start sites were predicted at the following website: http://www.fruitfly.org/seq_tools/promoter.html. The PCR product (1510 bp) was introduced into the pCAMBIA-1391 vector (Fig. S2) for fusion with the GUS reporter gene, yielding the transformation vector *OPR3p::GUS*. The ORF sequence of *CsOPR3* was isolated using the primers provided in Table S1 and inserted into the binary vector pCAMBIA1301 (Fig. S3), in which expression was driven by the cauliflower mosaic virus 35S promoter, yielding the overexpression vector *35S::OPR3*. The *OPR3p::GUS* and *35S::OPR3* vectors were inserted into the *Arabidopsis* WT and *opr3* mutant plants, respectively, using the *Agrobacterium*-mediated floral-dipping method^61^. Homozygous T2 plants were used for analysis.

### Southern blot analysis

Genomic DNA of *Arabidopsis* was isolated from fresh leaves using a plant genomic DNA kit (Tiangen, China). Southern blot analysis was carried out using the method of Xin et al.^12^, in which the GUS reporter gene was used as the probe. The GUS probe was obtained by PCR amplification using the primers listed in supplementary Table S1.

### Quantitative GUS activity assay and histochemical staining

Young leaves of 20-day-old homozygous T2 *Arabidopsis* seedlings subjected to different treatments were used for quantitative GUS analysis following the method of Xin et al.^12^. The amount of 4-methylumbel-liferone (4-MU) was detected by Skanlt RE for Varioskan Flash (Thermo, USA). For histochemical staining, 15-day-old *Arabidopsis* leaves were immersed in GUS staining solution (100 mM sodium phosphate, pH 7.0, 1 mM EDTA, 0.1% Triton X-100, and 1 mM X-Gluc) and incubated at 37 °C for 10 h. Chlorophyll in the samples was cleared with 70% ethanol.

### Cell death assay

Tea leaf disease symptoms caused by *C. fructicola* were assayed using trypan blue staining following the method of Li et al.^37^. The infected and control tea leaves were added to the lactophenol-trypan blue solution (10 ml of lactic acid, 10 ml of glycerol, 10 g of phenol, and 10 mg of trypan blue, dissolved in 10 ml of distilled water), boiled for 10 min, and destained by using chloral hydrate solution (25 g of chloral hydrate dissolved in 10 ml of distilled water) for 12 h. For cell death evaluation, the typical phenotypes were photographed using an optical microscope (Leica, Germany).

### JA, JA-Ile, SA, ABA, and H_2_O_2_ analysis

Freeze-ground tea or *Arabidopsis* leaf samples (50 mg) were mixed with 1 ml of ethyl acetate containing labeled internal standards. The homogenate was then centrifuged at 12,000*g* for 10 min at 4 °C, and the supernatants were evaporated at 30 °C until dryness. The extracts were dissolved in 200 μl of 70% MeOH and used for analysis. The contents of JA, JA-Ile, SA, and ABA were quantified by high-performance liquid chromatography–tandem MS using ^13^C_2_-JA, ^13^C_2_-JA-Ile, D_4_-SA, and D_6_-ABA as the internal standards, according to the method of Wu et al.^62^. For H_2_O_2_ analysis, 50 mg of a frozen powdered tea sample was added to 1 ml of deionized water, followed by shaking for 10 min. The homogenates were centrifuged at 12,000*g* for 10 min at 4 °C. The supernatants were retained for H_2_O_2_ analysis. H_2_O_2_ contents were measured following the method of Lou and Baldwin^63^. The localization of H_2_O_2_ production was detected by using DAB as described by Xin et al.^13^.

### Assay of defense enzyme activity

Tea leaves used for enzyme activity analysis were subjected to the following treatments: Con, Nerolidol, TLH, and Nerolidol + TLH. For the Nerolidol treatment, tea plants were exposed to (*E*)-nerolidol for 0.5 h and ventilated for 0.5 h. After 12 h, the samples were collected for measurement. For the TLH treatment, tea leaves were collected after12 h of TLH infestation. For the Nerolidol + TLH treatment, tea plants were exposed to (*E*)-nerolidol for 0.5 h and ventilated for 0.5 h, and the leaves were then treated with TLH for 12 h. Each treatment was performed with five replicates. Leaf samples (50 mg) were ground to a powder in liquid nitrogen and mixed with 1 ml of 0.2 mol l^−1^ SPB solution (pH 5.6) containing 50 mg polyvinylpolypyrrolidone. The slurry was centrifuged at 12,000*g* for 15 min at 4 °C, and the resulting supernatant was saved for analysis. PPO activity was estimated as described by Xin et al.^64^. Chitinase activity was calculated by using N-acetamino-glucose produced from colloidal chitin^65^. PAL activity was measured as described by Li et al.^66^ with l-phenylalanine as the substrate.

### Measurement of lignin and callose content

The contents of lignin and callose were quantified in tea plants subjected to the following treatments: Con, Nerolidol, Cfr, and Nerolidol + Cfr. For the Nerolidol treatment, tea plants were exposed to (*E*)-nerolidol for 0.5 h, followed by ventilation for 0.5 h. After 24 h, the samples were collected for measurement. For the Cfr treatment, tea leaves were harvested 24 h after *C. fructicola* infection. For the Nerolidol + Cfr treatment, tea plants were exposed to (*E*)-nerolidol for 0.5 h, followed by ventilation for 0.5 h, and were then infested with Cfr for 24 h. Each treatment was performed with five biological replicates. The callose in tea leaves was extracted and measured using the method of Liu et al.^43^. The callose staining assay was evaluated according to the method of Zhang et al.^67^. Lignin contents were measured quantitatively as described by Zeng et al.^68^. The absorbance was measured at 280 nm in a spectrophotometer. Lignin content was measured on a fresh weight basis.

### TLH preference measurement

To assess the feeding and oviposition preferences of TLH, two tea branches (one control plant vs. one (*E*)-nerolidol-treated plant) were covered in a glass cylinder. Each cylinder received 15 gravid TLH females. The number of TLH individuals on each branch was recorded at 1, 3, 6, 12, 24 and 48 h after TLH release. Three days after the experiment, TLHs were removed, and the eggs on the plant were recorded under a microscope. The experiment was conducted with six replicates. The survival rates of TLH nymphs on control or (*E*)-nerolidol-treated plants were also determined. Fifty TLH nymphs were placed on one tea plant that was confined within a square plastic cage. The numbers of surviving TLHs on each plant were recorded 72 h after the initiation of the experiment. The experiment was replicated six times. The honeydew excreted from one female TLH adult was collected in a small parafilm bag (6 cm × 5 cm), that was fixed on the tea leaves. The amount of honeydew was weighed 24 h after the experiment. Control and (*E*)-nerolidol-treated tea plants were used. The experiment was replicated 15 times.

### Statistical analysis

Statistical analysis was conducted with Statistica (SAS Institute, USA). The differences in the levels of GUS activity and defense-related compound contents were analyzed by analysis of variance followed by Duncan’s multiple-range test. Student’s *t* test was used for comparing two treatments.

## Supplementary information

Supplementary Materials
