# Supplementary material for: (E)-Nerolidol is a volatile signal that induces defenses against insects and pathogens in tea plants
Source: Hortic Res. 2020 Apr 1;7:52. doi: 10.1038/s41438-020-0275-7 (PMC7109047; doi:10.1038/s41438-020-0275-7)
Supplement: Supplementary file 1 — Supplementary Materials [file 41438_2020_275_MOESM1_ESM.docx]

**Supplementary Figure 1** Sequence of the 1.51kb *CsOPR3* promoter. The “A” marked with red indicates the transcriptional start site of *CsOPR3.*


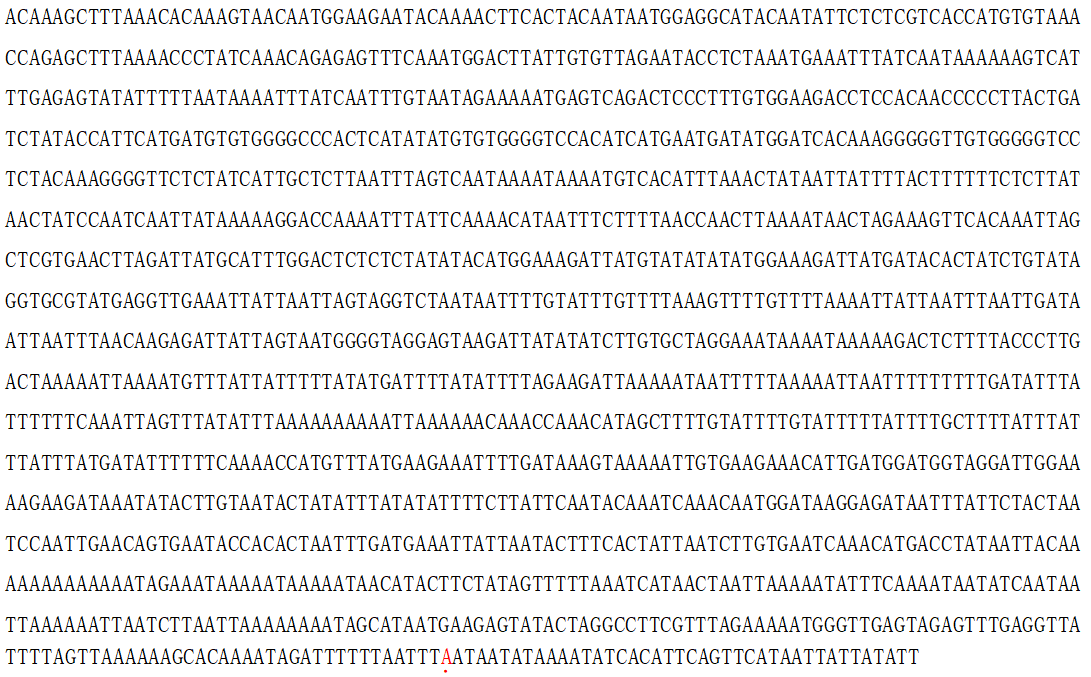


**Supplementary Figure 2** Schematic of the expression vectors pCAMBIA1301.


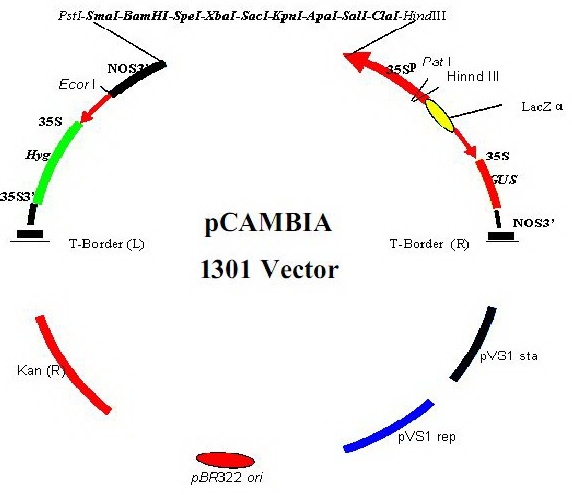


**Supplementary Figure 3** Schematic of the expression vectors pCAMBIA1391.

**
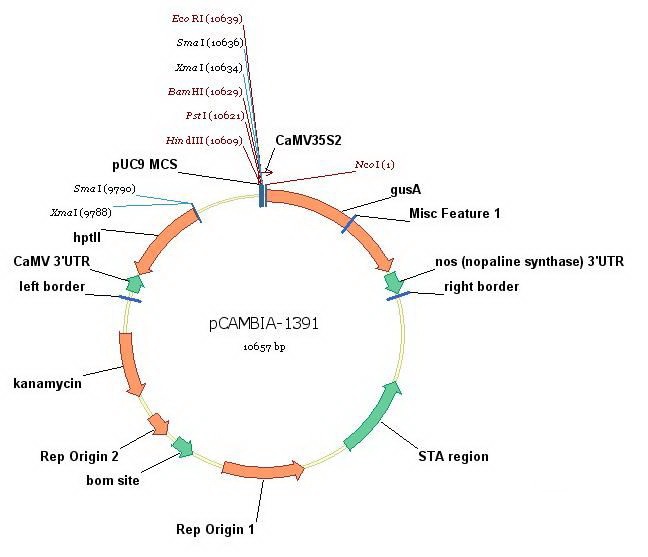
**

**Supplementary Table 1** The sequences of primers used in this study.

| Gene | Primer | Function | Sequence (5’-3’) |
| --- | --- | --- | --- |
| *CsOPR3* | F1 | Promoter-Forward | ACAAAGCTTTAAACACAAAGT |
|  | R1 | Promoter-Reverse | AATATAATAATTATGAACTG |
|  | F2 | qRT-PCR- Forward | CGATCAACAGCCGGTGGATTT |
|  | R2 | qRT-PCR- Reverse | GCGTGGACAGCATCAACCAC |
|  | F3 | Recombinant - Forward | TAGAATTCATGGCGGAGACGAGGTCG |
|  | R3 | Recombinant - Reverse | AGTCGACGGCGTGAAAATGGTCCAT |
|  | F4 | Overexpression- Forward | AGTCGACTTCATCCGATCACTTGTA |
|  | R4 | Overexpression- Reverse | ATTCTAGACACACGCGTGATCATGAC |
| GUS | F5 | Probe- Forward | GCAACTGGACAAGGCACT |
|  | R5 | Probe- Reverse | GCGTCGCAGAACATTACA |
| *GADPH* | F6 | qRT-PCR- Forward | ATACCACGTCATCCTCGGT |
|  | R6 | qRT-PCR- Reverse | ACTTATGATGAAATCAAAGCTGC |

**Supplementary Table S2:** List of the elements detected in 1.5kb *CsOPR3* promoter.

Factor or Site Name Loc.(Str.) Signal Sequence SITE #

-10PEHVPSBD 31 (-) TATTCT [S000392](https://sogo.dna.affrc.go.jp/cgi-bin/sogo.cgi?sid=&pj=640&action=newPlaceSite&site=S000392)

-10PEHVPSBD 69 (+) TATTCT [S000392](https://sogo.dna.affrc.go.jp/cgi-bin/sogo.cgi?sid=&pj=640&action=newPlaceSite&site=S000392)

-10PEHVPSBD 146 (-) TATTCT [S000392](https://sogo.dna.affrc.go.jp/cgi-bin/sogo.cgi?sid=&pj=640&action=newPlaceSite&site=S000392)

-10PEHVPSBD 1186 (+) TATTCT [S000392](https://sogo.dna.affrc.go.jp/cgi-bin/sogo.cgi?sid=&pj=640&action=newPlaceSite&site=S000392)

AACACOREOSGLUB1 960 (+) AACAAAC [S000353](https://sogo.dna.affrc.go.jp/cgi-bin/sogo.cgi?sid=&pj=640&action=newPlaceSite&site=S000353)

AMYBOX2 1168 (-) TATCCAT [S000021](https://sogo.dna.affrc.go.jp/cgi-bin/sogo.cgi?sid=&pj=640&action=newPlaceSite&site=S000021)

ANAERO1CONSENSUS 694 (-) AAACAAA [S000477](https://sogo.dna.affrc.go.jp/cgi-bin/sogo.cgi?sid=&pj=640&action=newPlaceSite&site=S000477)

ANAERO1CONSENSUS 707 (-) AAACAAA [S000477](https://sogo.dna.affrc.go.jp/cgi-bin/sogo.cgi?sid=&pj=640&action=newPlaceSite&site=S000477)

ANAERO1CONSENSUS 959 (+) AAACAAA [S000477](https://sogo.dna.affrc.go.jp/cgi-bin/sogo.cgi?sid=&pj=640&action=newPlaceSite&site=S000477)

ARR1AT 469 (-) NGATT [S000454](https://sogo.dna.affrc.go.jp/cgi-bin/sogo.cgi?sid=&pj=640&action=newPlaceSite&site=S000454)

ARR1AT 564 (+) NGATT [S000454](https://sogo.dna.affrc.go.jp/cgi-bin/sogo.cgi?sid=&pj=640&action=newPlaceSite&site=S000454)

ARR1AT 600 (+) NGATT [S000454](https://sogo.dna.affrc.go.jp/cgi-bin/sogo.cgi?sid=&pj=640&action=newPlaceSite&site=S000454)

ARR1AT 621 (+) NGATT [S000454](https://sogo.dna.affrc.go.jp/cgi-bin/sogo.cgi?sid=&pj=640&action=newPlaceSite&site=S000454)

ARR1AT 751 (+) NGATT [S000454](https://sogo.dna.affrc.go.jp/cgi-bin/sogo.cgi?sid=&pj=640&action=newPlaceSite&site=S000454)

ARR1AT 777 (+) NGATT [S000454](https://sogo.dna.affrc.go.jp/cgi-bin/sogo.cgi?sid=&pj=640&action=newPlaceSite&site=S000454)

ARR1AT 860 (+) NGATT [S000454](https://sogo.dna.affrc.go.jp/cgi-bin/sogo.cgi?sid=&pj=640&action=newPlaceSite&site=S000454)

ARR1AT 877 (+) NGATT [S000454](https://sogo.dna.affrc.go.jp/cgi-bin/sogo.cgi?sid=&pj=640&action=newPlaceSite&site=S000454)

ARR1AT 1096 (+) NGATT [S000454](https://sogo.dna.affrc.go.jp/cgi-bin/sogo.cgi?sid=&pj=640&action=newPlaceSite&site=S000454)

ARR1AT 1159 (-) NGATT [S000454](https://sogo.dna.affrc.go.jp/cgi-bin/sogo.cgi?sid=&pj=640&action=newPlaceSite&site=S000454)

ARR1AT 1195 (-) NGATT [S000454](https://sogo.dna.affrc.go.jp/cgi-bin/sogo.cgi?sid=&pj=640&action=newPlaceSite&site=S000454)

ARR1AT 1255 (-) NGATT [S000454](https://sogo.dna.affrc.go.jp/cgi-bin/sogo.cgi?sid=&pj=640&action=newPlaceSite&site=S000454)

ARR1AT 1264 (-) NGATT [S000454](https://sogo.dna.affrc.go.jp/cgi-bin/sogo.cgi?sid=&pj=640&action=newPlaceSite&site=S000454)

ARR1AT 1340 (-) NGATT [S000454](https://sogo.dna.affrc.go.jp/cgi-bin/sogo.cgi?sid=&pj=640&action=newPlaceSite&site=S000454)

ARR1AT 1391 (-) NGATT [S000454](https://sogo.dna.affrc.go.jp/cgi-bin/sogo.cgi?sid=&pj=640&action=newPlaceSite&site=S000454)

ARR1AT 1496 (+) NGATT [S000454](https://sogo.dna.affrc.go.jp/cgi-bin/sogo.cgi?sid=&pj=640&action=newPlaceSite&site=S000454)

ASF1MOTIFCAMV 77 (-) TGACG [S000024](https://sogo.dna.affrc.go.jp/cgi-bin/sogo.cgi?sid=&pj=640&action=newPlaceSite&site=S000024)

BIHD1OS 420 (+) TGTCA [S000498](https://sogo.dna.affrc.go.jp/cgi-bin/sogo.cgi?sid=&pj=640&action=newPlaceSite&site=S000498)

BOXIINTPATPB 222 (+) ATAGAA [S000296](https://sogo.dna.affrc.go.jp/cgi-bin/sogo.cgi?sid=&pj=640&action=newPlaceSite&site=S000296)

BOXIINTPATPB 1299 (+) ATAGAA [S000296](https://sogo.dna.affrc.go.jp/cgi-bin/sogo.cgi?sid=&pj=640&action=newPlaceSite&site=S000296)

BOXIINTPATPB 1326 (-) ATAGAA [S000296](https://sogo.dna.affrc.go.jp/cgi-bin/sogo.cgi?sid=&pj=640&action=newPlaceSite&site=S000296)

BOXLCOREDCPAL 1088 (-) ACCWWCC [S000492](https://sogo.dna.affrc.go.jp/cgi-bin/sogo.cgi?sid=&pj=640&action=newPlaceSite&site=S000492)

CAATBOX1 24 (+) CAAT [S000028](https://sogo.dna.affrc.go.jp/cgi-bin/sogo.cgi?sid=&pj=640&action=newPlaceSite&site=S000028)

CAATBOX1 50 (+) CAAT [S000028](https://sogo.dna.affrc.go.jp/cgi-bin/sogo.cgi?sid=&pj=640&action=newPlaceSite&site=S000028)

CAATBOX1 66 (+) CAAT [S000028](https://sogo.dna.affrc.go.jp/cgi-bin/sogo.cgi?sid=&pj=640&action=newPlaceSite&site=S000028)

CAATBOX1 138 (-) CAAT [S000028](https://sogo.dna.affrc.go.jp/cgi-bin/sogo.cgi?sid=&pj=640&action=newPlaceSite&site=S000028)

CAATBOX1 170 (+) CAAT [S000028](https://sogo.dna.affrc.go.jp/cgi-bin/sogo.cgi?sid=&pj=640&action=newPlaceSite&site=S000028)

CAATBOX1 213 (+) CAAT [S000028](https://sogo.dna.affrc.go.jp/cgi-bin/sogo.cgi?sid=&pj=640&action=newPlaceSite&site=S000028)

CAATBOX1 390 (-) CAAT [S000028](https://sogo.dna.affrc.go.jp/cgi-bin/sogo.cgi?sid=&pj=640&action=newPlaceSite&site=S000028)

CAATBOX1 407 (+) CAAT [S000028](https://sogo.dna.affrc.go.jp/cgi-bin/sogo.cgi?sid=&pj=640&action=newPlaceSite&site=S000028)

CAATBOX1 468 (+) CAAT [S000028](https://sogo.dna.affrc.go.jp/cgi-bin/sogo.cgi?sid=&pj=640&action=newPlaceSite&site=S000028)

CAATBOX1 472 (+) CAAT [S000028](https://sogo.dna.affrc.go.jp/cgi-bin/sogo.cgi?sid=&pj=640&action=newPlaceSite&site=S000028)

CAATBOX1 730 (-) CAAT [S000028](https://sogo.dna.affrc.go.jp/cgi-bin/sogo.cgi?sid=&pj=640&action=newPlaceSite&site=S000028)

CAATBOX1 1069 (-) CAAT [S000028](https://sogo.dna.affrc.go.jp/cgi-bin/sogo.cgi?sid=&pj=640&action=newPlaceSite&site=S000028)

CAATBOX1 1082 (-) CAAT [S000028](https://sogo.dna.affrc.go.jp/cgi-bin/sogo.cgi?sid=&pj=640&action=newPlaceSite&site=S000028)

CAATBOX1 1098 (-) CAAT [S000028](https://sogo.dna.affrc.go.jp/cgi-bin/sogo.cgi?sid=&pj=640&action=newPlaceSite&site=S000028)

CAATBOX1 1152 (+) CAAT [S000028](https://sogo.dna.affrc.go.jp/cgi-bin/sogo.cgi?sid=&pj=640&action=newPlaceSite&site=S000028)

CAATBOX1 1166 (+) CAAT [S000028](https://sogo.dna.affrc.go.jp/cgi-bin/sogo.cgi?sid=&pj=640&action=newPlaceSite&site=S000028)

CAATBOX1 1199 (+) CAAT [S000028](https://sogo.dna.affrc.go.jp/cgi-bin/sogo.cgi?sid=&pj=640&action=newPlaceSite&site=S000028)

CAATBOX1 1201 (-) CAAT [S000028](https://sogo.dna.affrc.go.jp/cgi-bin/sogo.cgi?sid=&pj=640&action=newPlaceSite&site=S000028)

CAATBOX1 1375 (+) CAAT [S000028](https://sogo.dna.affrc.go.jp/cgi-bin/sogo.cgi?sid=&pj=640&action=newPlaceSite&site=S000028)

CACTFTPPCA1 19 (-) YACT [S000449](https://sogo.dna.affrc.go.jp/cgi-bin/sogo.cgi?sid=&pj=640&action=newPlaceSite&site=S000449)

CACTFTPPCA1 45 (+) YACT [S000449](https://sogo.dna.affrc.go.jp/cgi-bin/sogo.cgi?sid=&pj=640&action=newPlaceSite&site=S000449)

CACTFTPPCA1 190 (-) YACT [S000449](https://sogo.dna.affrc.go.jp/cgi-bin/sogo.cgi?sid=&pj=640&action=newPlaceSite&site=S000449)

CACTFTPPCA1 271 (+) YACT [S000449](https://sogo.dna.affrc.go.jp/cgi-bin/sogo.cgi?sid=&pj=640&action=newPlaceSite&site=S000449)

CACTFTPPCA1 304 (+) YACT [S000449](https://sogo.dna.affrc.go.jp/cgi-bin/sogo.cgi?sid=&pj=640&action=newPlaceSite&site=S000449)

CACTFTPPCA1 445 (+) YACT [S000449](https://sogo.dna.affrc.go.jp/cgi-bin/sogo.cgi?sid=&pj=640&action=newPlaceSite&site=S000449)

CACTFTPPCA1 632 (+) YACT [S000449](https://sogo.dna.affrc.go.jp/cgi-bin/sogo.cgi?sid=&pj=640&action=newPlaceSite&site=S000449)

CACTFTPPCA1 673 (-) YACT [S000449](https://sogo.dna.affrc.go.jp/cgi-bin/sogo.cgi?sid=&pj=640&action=newPlaceSite&site=S000449)

CACTFTPPCA1 759 (-) YACT [S000449](https://sogo.dna.affrc.go.jp/cgi-bin/sogo.cgi?sid=&pj=640&action=newPlaceSite&site=S000449)

CACTFTPPCA1 773 (-) YACT [S000449](https://sogo.dna.affrc.go.jp/cgi-bin/sogo.cgi?sid=&pj=640&action=newPlaceSite&site=S000449)

CACTFTPPCA1 1062 (-) YACT [S000449](https://sogo.dna.affrc.go.jp/cgi-bin/sogo.cgi?sid=&pj=640&action=newPlaceSite&site=S000449)

CACTFTPPCA1 1118 (+) YACT [S000449](https://sogo.dna.affrc.go.jp/cgi-bin/sogo.cgi?sid=&pj=640&action=newPlaceSite&site=S000449)

CACTFTPPCA1 1127 (+) YACT [S000449](https://sogo.dna.affrc.go.jp/cgi-bin/sogo.cgi?sid=&pj=640&action=newPlaceSite&site=S000449)

CACTFTPPCA1 1191 (+) YACT [S000449](https://sogo.dna.affrc.go.jp/cgi-bin/sogo.cgi?sid=&pj=640&action=newPlaceSite&site=S000449)

CACTFTPPCA1 1208 (-) YACT [S000449](https://sogo.dna.affrc.go.jp/cgi-bin/sogo.cgi?sid=&pj=640&action=newPlaceSite&site=S000449)

CACTFTPPCA1 1219 (+) YACT [S000449](https://sogo.dna.affrc.go.jp/cgi-bin/sogo.cgi?sid=&pj=640&action=newPlaceSite&site=S000449)

CACTFTPPCA1 1242 (+) YACT [S000449](https://sogo.dna.affrc.go.jp/cgi-bin/sogo.cgi?sid=&pj=640&action=newPlaceSite&site=S000449)

CACTFTPPCA1 1248 (+) YACT [S000449](https://sogo.dna.affrc.go.jp/cgi-bin/sogo.cgi?sid=&pj=640&action=newPlaceSite&site=S000449)

CACTFTPPCA1 1323 (+) YACT [S000449](https://sogo.dna.affrc.go.jp/cgi-bin/sogo.cgi?sid=&pj=640&action=newPlaceSite&site=S000449)

CACTFTPPCA1 1422 (-) YACT [S000449](https://sogo.dna.affrc.go.jp/cgi-bin/sogo.cgi?sid=&pj=640&action=newPlaceSite&site=S000449)

CACTFTPPCA1 1426 (+) YACT [S000449](https://sogo.dna.affrc.go.jp/cgi-bin/sogo.cgi?sid=&pj=640&action=newPlaceSite&site=S000449)

CACTFTPPCA1 1456 (-) YACT [S000449](https://sogo.dna.affrc.go.jp/cgi-bin/sogo.cgi?sid=&pj=640&action=newPlaceSite&site=S000449)

CANBNNAPA 141 (-) CNAACAC [S000148](https://sogo.dna.affrc.go.jp/cgi-bin/sogo.cgi?sid=&pj=640&action=newPlaceSite&site=S000148)

CARGCW8GAT 396 (-) CWWWWWWWWG [S000431](https://sogo.dna.affrc.go.jp/cgi-bin/sogo.cgi?sid=&pj=640&action=newPlaceSite&site=S000431)

CARGCW8GAT 396 (+) CWWWWWWWWG [S000431](https://sogo.dna.affrc.go.jp/cgi-bin/sogo.cgi?sid=&pj=640&action=newPlaceSite&site=S000431)

CCA1ATLHCB1 1496 (-) AAMAATCT [S000149](https://sogo.dna.affrc.go.jp/cgi-bin/sogo.cgi?sid=&pj=640&action=newPlaceSite&site=S000149)

CCAATBOX1 467 (+) CCAAT [S000030](https://sogo.dna.affrc.go.jp/cgi-bin/sogo.cgi?sid=&pj=640&action=newPlaceSite&site=S000030)

CCAATBOX1 1098 (-) CCAAT [S000030](https://sogo.dna.affrc.go.jp/cgi-bin/sogo.cgi?sid=&pj=640&action=newPlaceSite&site=S000030)

CCAATBOX1 1198 (+) CCAAT [S000030](https://sogo.dna.affrc.go.jp/cgi-bin/sogo.cgi?sid=&pj=640&action=newPlaceSite&site=S000030)

CPBCSPOR 680 (-) TATTAG [S000491](https://sogo.dna.affrc.go.jp/cgi-bin/sogo.cgi?sid=&pj=640&action=newPlaceSite&site=S000491)

CPBCSPOR 755 (+) TATTAG [S000491](https://sogo.dna.affrc.go.jp/cgi-bin/sogo.cgi?sid=&pj=640&action=newPlaceSite&site=S000491)

DOFCOREZM 3 (+) AAAG [S000265](https://sogo.dna.affrc.go.jp/cgi-bin/sogo.cgi?sid=&pj=640&action=newPlaceSite&site=S000265)

DOFCOREZM 7 (-) AAAG [S000265](https://sogo.dna.affrc.go.jp/cgi-bin/sogo.cgi?sid=&pj=640&action=newPlaceSite&site=S000265)

DOFCOREZM 17 (+) AAAG [S000265](https://sogo.dna.affrc.go.jp/cgi-bin/sogo.cgi?sid=&pj=640&action=newPlaceSite&site=S000265)

DOFCOREZM 99 (-) AAAG [S000265](https://sogo.dna.affrc.go.jp/cgi-bin/sogo.cgi?sid=&pj=640&action=newPlaceSite&site=S000265)

DOFCOREZM 177 (+) AAAG [S000265](https://sogo.dna.affrc.go.jp/cgi-bin/sogo.cgi?sid=&pj=640&action=newPlaceSite&site=S000265)

DOFCOREZM 244 (-) AAAG [S000265](https://sogo.dna.affrc.go.jp/cgi-bin/sogo.cgi?sid=&pj=640&action=newPlaceSite&site=S000265)

DOFCOREZM 349 (+) AAAG [S000265](https://sogo.dna.affrc.go.jp/cgi-bin/sogo.cgi?sid=&pj=640&action=newPlaceSite&site=S000265)

DOFCOREZM 374 (+) AAAG [S000265](https://sogo.dna.affrc.go.jp/cgi-bin/sogo.cgi?sid=&pj=640&action=newPlaceSite&site=S000265)

DOFCOREZM 447 (-) AAAG [S000265](https://sogo.dna.affrc.go.jp/cgi-bin/sogo.cgi?sid=&pj=640&action=newPlaceSite&site=S000265)

DOFCOREZM 481 (+) AAAG [S000265](https://sogo.dna.affrc.go.jp/cgi-bin/sogo.cgi?sid=&pj=640&action=newPlaceSite&site=S000265)

DOFCOREZM 512 (-) AAAG [S000265](https://sogo.dna.affrc.go.jp/cgi-bin/sogo.cgi?sid=&pj=640&action=newPlaceSite&site=S000265)

DOFCOREZM 537 (+) AAAG [S000265](https://sogo.dna.affrc.go.jp/cgi-bin/sogo.cgi?sid=&pj=640&action=newPlaceSite&site=S000265)

DOFCOREZM 598 (+) AAAG [S000265](https://sogo.dna.affrc.go.jp/cgi-bin/sogo.cgi?sid=&pj=640&action=newPlaceSite&site=S000265)

DOFCOREZM 619 (+) AAAG [S000265](https://sogo.dna.affrc.go.jp/cgi-bin/sogo.cgi?sid=&pj=640&action=newPlaceSite&site=S000265)

DOFCOREZM 702 (+) AAAG [S000265](https://sogo.dna.affrc.go.jp/cgi-bin/sogo.cgi?sid=&pj=640&action=newPlaceSite&site=S000265)

DOFCOREZM 810 (+) AAAG [S000265](https://sogo.dna.affrc.go.jp/cgi-bin/sogo.cgi?sid=&pj=640&action=newPlaceSite&site=S000265)

DOFCOREZM 817 (-) AAAG [S000265](https://sogo.dna.affrc.go.jp/cgi-bin/sogo.cgi?sid=&pj=640&action=newPlaceSite&site=S000265)

DOFCOREZM 976 (-) AAAG [S000265](https://sogo.dna.affrc.go.jp/cgi-bin/sogo.cgi?sid=&pj=640&action=newPlaceSite&site=S000265)

DOFCOREZM 1002 (-) AAAG [S000265](https://sogo.dna.affrc.go.jp/cgi-bin/sogo.cgi?sid=&pj=640&action=newPlaceSite&site=S000265)

DOFCOREZM 1060 (+) AAAG [S000265](https://sogo.dna.affrc.go.jp/cgi-bin/sogo.cgi?sid=&pj=640&action=newPlaceSite&site=S000265)

DOFCOREZM 1104 (+) AAAG [S000265](https://sogo.dna.affrc.go.jp/cgi-bin/sogo.cgi?sid=&pj=640&action=newPlaceSite&site=S000265)

DOFCOREZM 1244 (-) AAAG [S000265](https://sogo.dna.affrc.go.jp/cgi-bin/sogo.cgi?sid=&pj=640&action=newPlaceSite&site=S000265)

DOFCOREZM 1484 (+) AAAG [S000265](https://sogo.dna.affrc.go.jp/cgi-bin/sogo.cgi?sid=&pj=640&action=newPlaceSite&site=S000265)

DPBFCOREDCDC3 83 (-) ACACNNG [S000292](https://sogo.dna.affrc.go.jp/cgi-bin/sogo.cgi?sid=&pj=640&action=newPlaceSite&site=S000292)

EBOXBNNAPA 83 (-) CANNTG [S000144](https://sogo.dna.affrc.go.jp/cgi-bin/sogo.cgi?sid=&pj=640&action=newPlaceSite&site=S000144)

EBOXBNNAPA 83 (+) CANNTG [S000144](https://sogo.dna.affrc.go.jp/cgi-bin/sogo.cgi?sid=&pj=640&action=newPlaceSite&site=S000144)

EBOXBNNAPA 127 (-) CANNTG [S000144](https://sogo.dna.affrc.go.jp/cgi-bin/sogo.cgi?sid=&pj=640&action=newPlaceSite&site=S000144)

EBOXBNNAPA 127 (+) CANNTG [S000144](https://sogo.dna.affrc.go.jp/cgi-bin/sogo.cgi?sid=&pj=640&action=newPlaceSite&site=S000144)

EBOXBNNAPA 182 (-) CANNTG [S000144](https://sogo.dna.affrc.go.jp/cgi-bin/sogo.cgi?sid=&pj=640&action=newPlaceSite&site=S000144)

EBOXBNNAPA 182 (+) CANNTG [S000144](https://sogo.dna.affrc.go.jp/cgi-bin/sogo.cgi?sid=&pj=640&action=newPlaceSite&site=S000144)

EBOXBNNAPA 572 (-) CANNTG [S000144](https://sogo.dna.affrc.go.jp/cgi-bin/sogo.cgi?sid=&pj=640&action=newPlaceSite&site=S000144)

EBOXBNNAPA 572 (+) CANNTG [S000144](https://sogo.dna.affrc.go.jp/cgi-bin/sogo.cgi?sid=&pj=640&action=newPlaceSite&site=S000144)

EBOXBNNAPA 1199 (-) CANNTG [S000144](https://sogo.dna.affrc.go.jp/cgi-bin/sogo.cgi?sid=&pj=640&action=newPlaceSite&site=S000144)

EBOXBNNAPA 1199 (+) CANNTG [S000144](https://sogo.dna.affrc.go.jp/cgi-bin/sogo.cgi?sid=&pj=640&action=newPlaceSite&site=S000144)

EECCRCAH1 121 (+) GANTTNC [S000494](https://sogo.dna.affrc.go.jp/cgi-bin/sogo.cgi?sid=&pj=640&action=newPlaceSite&site=S000494)

ERELEE4 1360 (+) AWTTCAAA [S000037](https://sogo.dna.affrc.go.jp/cgi-bin/sogo.cgi?sid=&pj=640&action=newPlaceSite&site=S000037)

GAREAT 744 (+) TAACAAR [S000439](https://sogo.dna.affrc.go.jp/cgi-bin/sogo.cgi?sid=&pj=640&action=newPlaceSite&site=S000439)

GATABOX 110 (-) GATA [S000039](https://sogo.dna.affrc.go.jp/cgi-bin/sogo.cgi?sid=&pj=640&action=newPlaceSite&site=S000039)

GATABOX 167 (-) GATA [S000039](https://sogo.dna.affrc.go.jp/cgi-bin/sogo.cgi?sid=&pj=640&action=newPlaceSite&site=S000039)

GATABOX 210 (-) GATA [S000039](https://sogo.dna.affrc.go.jp/cgi-bin/sogo.cgi?sid=&pj=640&action=newPlaceSite&site=S000039)

GATABOX 337 (+) GATA [S000039](https://sogo.dna.affrc.go.jp/cgi-bin/sogo.cgi?sid=&pj=640&action=newPlaceSite&site=S000039)

GATABOX 386 (-) GATA [S000039](https://sogo.dna.affrc.go.jp/cgi-bin/sogo.cgi?sid=&pj=640&action=newPlaceSite&site=S000039)

GATABOX 464 (-) GATA [S000039](https://sogo.dna.affrc.go.jp/cgi-bin/sogo.cgi?sid=&pj=640&action=newPlaceSite&site=S000039)

GATABOX 628 (+) GATA [S000039](https://sogo.dna.affrc.go.jp/cgi-bin/sogo.cgi?sid=&pj=640&action=newPlaceSite&site=S000039)

GATABOX 635 (-) GATA [S000039](https://sogo.dna.affrc.go.jp/cgi-bin/sogo.cgi?sid=&pj=640&action=newPlaceSite&site=S000039)

GATABOX 733 (+) GATA [S000039](https://sogo.dna.affrc.go.jp/cgi-bin/sogo.cgi?sid=&pj=640&action=newPlaceSite&site=S000039)

GATABOX 785 (-) GATA [S000039](https://sogo.dna.affrc.go.jp/cgi-bin/sogo.cgi?sid=&pj=640&action=newPlaceSite&site=S000039)

GATABOX 913 (+) GATA [S000039](https://sogo.dna.affrc.go.jp/cgi-bin/sogo.cgi?sid=&pj=640&action=newPlaceSite&site=S000039)

GATABOX 1021 (+) GATA [S000039](https://sogo.dna.affrc.go.jp/cgi-bin/sogo.cgi?sid=&pj=640&action=newPlaceSite&site=S000039)

GATABOX 1057 (+) GATA [S000039](https://sogo.dna.affrc.go.jp/cgi-bin/sogo.cgi?sid=&pj=640&action=newPlaceSite&site=S000039)

GATABOX 1110 (+) GATA [S000039](https://sogo.dna.affrc.go.jp/cgi-bin/sogo.cgi?sid=&pj=640&action=newPlaceSite&site=S000039)

GATABOX 1171 (+) GATA [S000039](https://sogo.dna.affrc.go.jp/cgi-bin/sogo.cgi?sid=&pj=640&action=newPlaceSite&site=S000039)

GATABOX 1179 (+) GATA [S000039](https://sogo.dna.affrc.go.jp/cgi-bin/sogo.cgi?sid=&pj=640&action=newPlaceSite&site=S000039)

GATABOX 1372 (-) GATA [S000039](https://sogo.dna.affrc.go.jp/cgi-bin/sogo.cgi?sid=&pj=640&action=newPlaceSite&site=S000039)

GATABOX 1522 (-) GATA [S000039](https://sogo.dna.affrc.go.jp/cgi-bin/sogo.cgi?sid=&pj=640&action=newPlaceSite&site=S000039)

GCN4OSGLUB1 231 (+) TGAGTCA [S000277](https://sogo.dna.affrc.go.jp/cgi-bin/sogo.cgi?sid=&pj=640&action=newPlaceSite&site=S000277)

GT1CONSENSUS 165 (-) GRWAAW [S000198](https://sogo.dna.affrc.go.jp/cgi-bin/sogo.cgi?sid=&pj=640&action=newPlaceSite&site=S000198)

GT1CONSENSUS 208 (-) GRWAAW [S000198](https://sogo.dna.affrc.go.jp/cgi-bin/sogo.cgi?sid=&pj=640&action=newPlaceSite&site=S000198)

GT1CONSENSUS 225 (+) GRWAAW [S000198](https://sogo.dna.affrc.go.jp/cgi-bin/sogo.cgi?sid=&pj=640&action=newPlaceSite&site=S000198)

GT1CONSENSUS 449 (-) GRWAAW [S000198](https://sogo.dna.affrc.go.jp/cgi-bin/sogo.cgi?sid=&pj=640&action=newPlaceSite&site=S000198)

GT1CONSENSUS 733 (+) GRWAAW [S000198](https://sogo.dna.affrc.go.jp/cgi-bin/sogo.cgi?sid=&pj=640&action=newPlaceSite&site=S000198)

GT1CONSENSUS 797 (+) GRWAAW [S000198](https://sogo.dna.affrc.go.jp/cgi-bin/sogo.cgi?sid=&pj=640&action=newPlaceSite&site=S000198)

GT1CONSENSUS 819 (-) GRWAAW [S000198](https://sogo.dna.affrc.go.jp/cgi-bin/sogo.cgi?sid=&pj=640&action=newPlaceSite&site=S000198)

GT1CONSENSUS 922 (-) GRWAAW [S000198](https://sogo.dna.affrc.go.jp/cgi-bin/sogo.cgi?sid=&pj=640&action=newPlaceSite&site=S000198)

GT1CONSENSUS 1026 (-) GRWAAW [S000198](https://sogo.dna.affrc.go.jp/cgi-bin/sogo.cgi?sid=&pj=640&action=newPlaceSite&site=S000198)

GT1CONSENSUS 1057 (+) GRWAAW [S000198](https://sogo.dna.affrc.go.jp/cgi-bin/sogo.cgi?sid=&pj=640&action=newPlaceSite&site=S000198)

GT1CONSENSUS 1101 (+) GRWAAW [S000198](https://sogo.dna.affrc.go.jp/cgi-bin/sogo.cgi?sid=&pj=640&action=newPlaceSite&site=S000198)

GT1CONSENSUS 1110 (+) GRWAAW [S000198](https://sogo.dna.affrc.go.jp/cgi-bin/sogo.cgi?sid=&pj=640&action=newPlaceSite&site=S000198)

GT1CONSENSUS 1141 (-) GRWAAW [S000198](https://sogo.dna.affrc.go.jp/cgi-bin/sogo.cgi?sid=&pj=640&action=newPlaceSite&site=S000198)

GT1CONSENSUS 1179 (+) GRWAAW [S000198](https://sogo.dna.affrc.go.jp/cgi-bin/sogo.cgi?sid=&pj=640&action=newPlaceSite&site=S000198)

GT1CONSENSUS 1443 (+) GRWAAW [S000198](https://sogo.dna.affrc.go.jp/cgi-bin/sogo.cgi?sid=&pj=640&action=newPlaceSite&site=S000198)

GT1CORE 515 (-) GGTTAA [S000125](https://sogo.dna.affrc.go.jp/cgi-bin/sogo.cgi?sid=&pj=640&action=newPlaceSite&site=S000125)

GT1GMSCAM4 225 (+) GAAAAA [S000453](https://sogo.dna.affrc.go.jp/cgi-bin/sogo.cgi?sid=&pj=640&action=newPlaceSite&site=S000453)

GT1GMSCAM4 449 (-) GAAAAA [S000453](https://sogo.dna.affrc.go.jp/cgi-bin/sogo.cgi?sid=&pj=640&action=newPlaceSite&site=S000453)

GT1GMSCAM4 922 (-) GAAAAA [S000453](https://sogo.dna.affrc.go.jp/cgi-bin/sogo.cgi?sid=&pj=640&action=newPlaceSite&site=S000453)

GT1GMSCAM4 1026 (-) GAAAAA [S000453](https://sogo.dna.affrc.go.jp/cgi-bin/sogo.cgi?sid=&pj=640&action=newPlaceSite&site=S000453)

GT1GMSCAM4 1443 (+) GAAAAA [S000453](https://sogo.dna.affrc.go.jp/cgi-bin/sogo.cgi?sid=&pj=640&action=newPlaceSite&site=S000453)

GTGANTG10 44 (-) GTGA [S000378](https://sogo.dna.affrc.go.jp/cgi-bin/sogo.cgi?sid=&pj=640&action=newPlaceSite&site=S000378)

GTGANTG10 79 (-) GTGA [S000378](https://sogo.dna.affrc.go.jp/cgi-bin/sogo.cgi?sid=&pj=640&action=newPlaceSite&site=S000378)

GTGANTG10 345 (-) GTGA [S000378](https://sogo.dna.affrc.go.jp/cgi-bin/sogo.cgi?sid=&pj=640&action=newPlaceSite&site=S000378)

GTGANTG10 422 (-) GTGA [S000378](https://sogo.dna.affrc.go.jp/cgi-bin/sogo.cgi?sid=&pj=640&action=newPlaceSite&site=S000378)

GTGANTG10 542 (-) GTGA [S000378](https://sogo.dna.affrc.go.jp/cgi-bin/sogo.cgi?sid=&pj=640&action=newPlaceSite&site=S000378)

GTGANTG10 556 (+) GTGA [S000378](https://sogo.dna.affrc.go.jp/cgi-bin/sogo.cgi?sid=&pj=640&action=newPlaceSite&site=S000378)

GTGANTG10 1072 (+) GTGA [S000378](https://sogo.dna.affrc.go.jp/cgi-bin/sogo.cgi?sid=&pj=640&action=newPlaceSite&site=S000378)

GTGANTG10 1209 (+) GTGA [S000378](https://sogo.dna.affrc.go.jp/cgi-bin/sogo.cgi?sid=&pj=640&action=newPlaceSite&site=S000378)

GTGANTG10 1247 (-) GTGA [S000378](https://sogo.dna.affrc.go.jp/cgi-bin/sogo.cgi?sid=&pj=640&action=newPlaceSite&site=S000378)

GTGANTG10 1261 (+) GTGA [S000378](https://sogo.dna.affrc.go.jp/cgi-bin/sogo.cgi?sid=&pj=640&action=newPlaceSite&site=S000378)

GTGANTG10 1524 (-) GTGA [S000378](https://sogo.dna.affrc.go.jp/cgi-bin/sogo.cgi?sid=&pj=640&action=newPlaceSite&site=S000378)

HDZIP2ATATHB2 1538 (-) TAATMATTA [S000373](https://sogo.dna.affrc.go.jp/cgi-bin/sogo.cgi?sid=&pj=640&action=newPlaceSite&site=S000373)

IBOX 1171 (+) GATAAG [S000124](https://sogo.dna.affrc.go.jp/cgi-bin/sogo.cgi?sid=&pj=640&action=newPlaceSite&site=S000124)

IBOXCORE 166 (-) GATAA [S000199](https://sogo.dna.affrc.go.jp/cgi-bin/sogo.cgi?sid=&pj=640&action=newPlaceSite&site=S000199)

IBOXCORE 209 (-) GATAA [S000199](https://sogo.dna.affrc.go.jp/cgi-bin/sogo.cgi?sid=&pj=640&action=newPlaceSite&site=S000199)

IBOXCORE 733 (+) GATAA [S000199](https://sogo.dna.affrc.go.jp/cgi-bin/sogo.cgi?sid=&pj=640&action=newPlaceSite&site=S000199)

IBOXCORE 1057 (+) GATAA [S000199](https://sogo.dna.affrc.go.jp/cgi-bin/sogo.cgi?sid=&pj=640&action=newPlaceSite&site=S000199)

IBOXCORE 1110 (+) GATAA [S000199](https://sogo.dna.affrc.go.jp/cgi-bin/sogo.cgi?sid=&pj=640&action=newPlaceSite&site=S000199)

IBOXCORE 1171 (+) GATAA [S000199](https://sogo.dna.affrc.go.jp/cgi-bin/sogo.cgi?sid=&pj=640&action=newPlaceSite&site=S000199)

IBOXCORE 1179 (+) GATAA [S000199](https://sogo.dna.affrc.go.jp/cgi-bin/sogo.cgi?sid=&pj=640&action=newPlaceSite&site=S000199)

IBOXCORENT 1171 (+) GATAAGR [S000424](https://sogo.dna.affrc.go.jp/cgi-bin/sogo.cgi?sid=&pj=640&action=newPlaceSite&site=S000424)

INRNTPSADB 227 (-) YTCANTYY [S000395](https://sogo.dna.affrc.go.jp/cgi-bin/sogo.cgi?sid=&pj=640&action=newPlaceSite&site=S000395)

INRNTPSADB 1529 (+) YTCANTYY [S000395](https://sogo.dna.affrc.go.jp/cgi-bin/sogo.cgi?sid=&pj=640&action=newPlaceSite&site=S000395)

LECPLEACS2 867 (-) TAAAATAT [S000465](https://sogo.dna.affrc.go.jp/cgi-bin/sogo.cgi?sid=&pj=640&action=newPlaceSite&site=S000465)

LECPLEACS2 1517 (+) TAAAATAT [S000465](https://sogo.dna.affrc.go.jp/cgi-bin/sogo.cgi?sid=&pj=640&action=newPlaceSite&site=S000465)

MARABOX1 1004 (-) AATAAAYAAA [S000063](https://sogo.dna.affrc.go.jp/cgi-bin/sogo.cgi?sid=&pj=640&action=newPlaceSite&site=S000063)

MARABOX1 1008 (-) AATAAAYAAA [S000063](https://sogo.dna.affrc.go.jp/cgi-bin/sogo.cgi?sid=&pj=640&action=newPlaceSite&site=S000063)

MARARS 863 (+) WTTTATRTTTW [S000064](https://sogo.dna.affrc.go.jp/cgi-bin/sogo.cgi?sid=&pj=640&action=newPlaceSite&site=S000064)

MARTBOX 408 (-) TTWTWTTWTT [S000067](https://sogo.dna.affrc.go.jp/cgi-bin/sogo.cgi?sid=&pj=640&action=newPlaceSite&site=S000067)

MARTBOX 800 (-) TTWTWTTWTT [S000067](https://sogo.dna.affrc.go.jp/cgi-bin/sogo.cgi?sid=&pj=640&action=newPlaceSite&site=S000067)

MARTBOX 944 (-) TTWTWTTWTT [S000067](https://sogo.dna.affrc.go.jp/cgi-bin/sogo.cgi?sid=&pj=640&action=newPlaceSite&site=S000067)

MARTBOX 1287 (-) TTWTWTTWTT [S000067](https://sogo.dna.affrc.go.jp/cgi-bin/sogo.cgi?sid=&pj=640&action=newPlaceSite&site=S000067)

MARTBOX 1288 (-) TTWTWTTWTT [S000067](https://sogo.dna.affrc.go.jp/cgi-bin/sogo.cgi?sid=&pj=640&action=newPlaceSite&site=S000067)

MARTBOX 1289 (-) TTWTWTTWTT [S000067](https://sogo.dna.affrc.go.jp/cgi-bin/sogo.cgi?sid=&pj=640&action=newPlaceSite&site=S000067)

MARTBOX 1290 (-) TTWTWTTWTT [S000067](https://sogo.dna.affrc.go.jp/cgi-bin/sogo.cgi?sid=&pj=640&action=newPlaceSite&site=S000067)

MARTBOX 1307 (-) TTWTWTTWTT [S000067](https://sogo.dna.affrc.go.jp/cgi-bin/sogo.cgi?sid=&pj=640&action=newPlaceSite&site=S000067)

MARTBOX 1510 (-) TTWTWTTWTT [S000067](https://sogo.dna.affrc.go.jp/cgi-bin/sogo.cgi?sid=&pj=640&action=newPlaceSite&site=S000067)

MYB1AT 90 (+) WAACCA [S000408](https://sogo.dna.affrc.go.jp/cgi-bin/sogo.cgi?sid=&pj=640&action=newPlaceSite&site=S000408)

MYB1AT 516 (+) WAACCA [S000408](https://sogo.dna.affrc.go.jp/cgi-bin/sogo.cgi?sid=&pj=640&action=newPlaceSite&site=S000408)

MYB1AT 963 (+) WAACCA [S000408](https://sogo.dna.affrc.go.jp/cgi-bin/sogo.cgi?sid=&pj=640&action=newPlaceSite&site=S000408)

MYB1AT 1033 (+) WAACCA [S000408](https://sogo.dna.affrc.go.jp/cgi-bin/sogo.cgi?sid=&pj=640&action=newPlaceSite&site=S000408)

MYBPLANT 964 (+) MACCWAMC [S000167](https://sogo.dna.affrc.go.jp/cgi-bin/sogo.cgi?sid=&pj=640&action=newPlaceSite&site=S000167)

MYBPZM 767 (-) CCWACC [S000179](https://sogo.dna.affrc.go.jp/cgi-bin/sogo.cgi?sid=&pj=640&action=newPlaceSite&site=S000179)

MYBPZM 1092 (-) CCWACC [S000179](https://sogo.dna.affrc.go.jp/cgi-bin/sogo.cgi?sid=&pj=640&action=newPlaceSite&site=S000179)

MYBST1 464 (-) GGATA [S000180](https://sogo.dna.affrc.go.jp/cgi-bin/sogo.cgi?sid=&pj=640&action=newPlaceSite&site=S000180)

MYBST1 1170 (+) GGATA [S000180](https://sogo.dna.affrc.go.jp/cgi-bin/sogo.cgi?sid=&pj=640&action=newPlaceSite&site=S000180)

MYCATERD1 83 (+) CATGTG [S000413](https://sogo.dna.affrc.go.jp/cgi-bin/sogo.cgi?sid=&pj=640&action=newPlaceSite&site=S000413)

MYCATRD22 83 (-) CACATG [S000174](https://sogo.dna.affrc.go.jp/cgi-bin/sogo.cgi?sid=&pj=640&action=newPlaceSite&site=S000174)

MYCCONSENSUSAT 83 (-) CANNTG [S000407](https://sogo.dna.affrc.go.jp/cgi-bin/sogo.cgi?sid=&pj=640&action=newPlaceSite&site=S000407)

MYCCONSENSUSAT 83 (+) CANNTG [S000407](https://sogo.dna.affrc.go.jp/cgi-bin/sogo.cgi?sid=&pj=640&action=newPlaceSite&site=S000407)

MYCCONSENSUSAT 127 (-) CANNTG [S000407](https://sogo.dna.affrc.go.jp/cgi-bin/sogo.cgi?sid=&pj=640&action=newPlaceSite&site=S000407)

MYCCONSENSUSAT 127 (+) CANNTG [S000407](https://sogo.dna.affrc.go.jp/cgi-bin/sogo.cgi?sid=&pj=640&action=newPlaceSite&site=S000407)

MYCCONSENSUSAT 182 (-) CANNTG [S000407](https://sogo.dna.affrc.go.jp/cgi-bin/sogo.cgi?sid=&pj=640&action=newPlaceSite&site=S000407)

MYCCONSENSUSAT 182 (+) CANNTG [S000407](https://sogo.dna.affrc.go.jp/cgi-bin/sogo.cgi?sid=&pj=640&action=newPlaceSite&site=S000407)

MYCCONSENSUSAT 572 (-) CANNTG [S000407](https://sogo.dna.affrc.go.jp/cgi-bin/sogo.cgi?sid=&pj=640&action=newPlaceSite&site=S000407)

MYCCONSENSUSAT 572 (+) CANNTG [S000407](https://sogo.dna.affrc.go.jp/cgi-bin/sogo.cgi?sid=&pj=640&action=newPlaceSite&site=S000407)

MYCCONSENSUSAT 1199 (-) CANNTG [S000407](https://sogo.dna.affrc.go.jp/cgi-bin/sogo.cgi?sid=&pj=640&action=newPlaceSite&site=S000407)

MYCCONSENSUSAT 1199 (+) CANNTG [S000407](https://sogo.dna.affrc.go.jp/cgi-bin/sogo.cgi?sid=&pj=640&action=newPlaceSite&site=S000407)

NAPINMOTIFBN 84 (-) TACACAT [S000070](https://sogo.dna.affrc.go.jp/cgi-bin/sogo.cgi?sid=&pj=640&action=newPlaceSite&site=S000070)

NODCON1GM 598 (+) AAAGAT [S000461](https://sogo.dna.affrc.go.jp/cgi-bin/sogo.cgi?sid=&pj=640&action=newPlaceSite&site=S000461)

NODCON1GM 619 (+) AAAGAT [S000461](https://sogo.dna.affrc.go.jp/cgi-bin/sogo.cgi?sid=&pj=640&action=newPlaceSite&site=S000461)

NODCON2GM 394 (+) CTCTT [S000462](https://sogo.dna.affrc.go.jp/cgi-bin/sogo.cgi?sid=&pj=640&action=newPlaceSite&site=S000462)

NODCON2GM 454 (+) CTCTT [S000462](https://sogo.dna.affrc.go.jp/cgi-bin/sogo.cgi?sid=&pj=640&action=newPlaceSite&site=S000462)

NODCON2GM 748 (-) CTCTT [S000462](https://sogo.dna.affrc.go.jp/cgi-bin/sogo.cgi?sid=&pj=640&action=newPlaceSite&site=S000462)

NODCON2GM 815 (+) CTCTT [S000462](https://sogo.dna.affrc.go.jp/cgi-bin/sogo.cgi?sid=&pj=640&action=newPlaceSite&site=S000462)

NODCON2GM 1419 (-) CTCTT [S000462](https://sogo.dna.affrc.go.jp/cgi-bin/sogo.cgi?sid=&pj=640&action=newPlaceSite&site=S000462)

NTBBF1ARROLB 701 (-) ACTTTA [S000273](https://sogo.dna.affrc.go.jp/cgi-bin/sogo.cgi?sid=&pj=640&action=newPlaceSite&site=S000273)

NTBBF1ARROLB 1059 (-) ACTTTA [S000273](https://sogo.dna.affrc.go.jp/cgi-bin/sogo.cgi?sid=&pj=640&action=newPlaceSite&site=S000273)

OSE1ROOTNODULE 598 (+) AAAGAT [S000467](https://sogo.dna.affrc.go.jp/cgi-bin/sogo.cgi?sid=&pj=640&action=newPlaceSite&site=S000467)

OSE1ROOTNODULE 619 (+) AAAGAT [S000467](https://sogo.dna.affrc.go.jp/cgi-bin/sogo.cgi?sid=&pj=640&action=newPlaceSite&site=S000467)

OSE2ROOTNODULE 394 (+) CTCTT [S000468](https://sogo.dna.affrc.go.jp/cgi-bin/sogo.cgi?sid=&pj=640&action=newPlaceSite&site=S000468)

OSE2ROOTNODULE 454 (+) CTCTT [S000468](https://sogo.dna.affrc.go.jp/cgi-bin/sogo.cgi?sid=&pj=640&action=newPlaceSite&site=S000468)

OSE2ROOTNODULE 748 (-) CTCTT [S000468](https://sogo.dna.affrc.go.jp/cgi-bin/sogo.cgi?sid=&pj=640&action=newPlaceSite&site=S000468)

OSE2ROOTNODULE 815 (+) CTCTT [S000468](https://sogo.dna.affrc.go.jp/cgi-bin/sogo.cgi?sid=&pj=640&action=newPlaceSite&site=S000468)

OSE2ROOTNODULE 1419 (-) CTCTT [S000468](https://sogo.dna.affrc.go.jp/cgi-bin/sogo.cgi?sid=&pj=640&action=newPlaceSite&site=S000468)

POLASIG1 171 (+) AATAAA [S000080](https://sogo.dna.affrc.go.jp/cgi-bin/sogo.cgi?sid=&pj=640&action=newPlaceSite&site=S000080)

POLASIG1 201 (+) AATAAA [S000080](https://sogo.dna.affrc.go.jp/cgi-bin/sogo.cgi?sid=&pj=640&action=newPlaceSite&site=S000080)

POLASIG1 408 (+) AATAAA [S000080](https://sogo.dna.affrc.go.jp/cgi-bin/sogo.cgi?sid=&pj=640&action=newPlaceSite&site=S000080)

POLASIG1 413 (+) AATAAA [S000080](https://sogo.dna.affrc.go.jp/cgi-bin/sogo.cgi?sid=&pj=640&action=newPlaceSite&site=S000080)

POLASIG1 493 (-) AATAAA [S000080](https://sogo.dna.affrc.go.jp/cgi-bin/sogo.cgi?sid=&pj=640&action=newPlaceSite&site=S000080)

POLASIG1 800 (+) AATAAA [S000080](https://sogo.dna.affrc.go.jp/cgi-bin/sogo.cgi?sid=&pj=640&action=newPlaceSite&site=S000080)

POLASIG1 805 (+) AATAAA [S000080](https://sogo.dna.affrc.go.jp/cgi-bin/sogo.cgi?sid=&pj=640&action=newPlaceSite&site=S000080)

POLASIG1 845 (-) AATAAA [S000080](https://sogo.dna.affrc.go.jp/cgi-bin/sogo.cgi?sid=&pj=640&action=newPlaceSite&site=S000080)

POLASIG1 917 (-) AATAAA [S000080](https://sogo.dna.affrc.go.jp/cgi-bin/sogo.cgi?sid=&pj=640&action=newPlaceSite&site=S000080)

POLASIG1 993 (-) AATAAA [S000080](https://sogo.dna.affrc.go.jp/cgi-bin/sogo.cgi?sid=&pj=640&action=newPlaceSite&site=S000080)

POLASIG1 1004 (-) AATAAA [S000080](https://sogo.dna.affrc.go.jp/cgi-bin/sogo.cgi?sid=&pj=640&action=newPlaceSite&site=S000080)

POLASIG1 1008 (-) AATAAA [S000080](https://sogo.dna.affrc.go.jp/cgi-bin/sogo.cgi?sid=&pj=640&action=newPlaceSite&site=S000080)

POLASIG1 1012 (-) AATAAA [S000080](https://sogo.dna.affrc.go.jp/cgi-bin/sogo.cgi?sid=&pj=640&action=newPlaceSite&site=S000080)

POLASIG1 1184 (-) AATAAA [S000080](https://sogo.dna.affrc.go.jp/cgi-bin/sogo.cgi?sid=&pj=640&action=newPlaceSite&site=S000080)

POLASIG1 1304 (+) AATAAA [S000080](https://sogo.dna.affrc.go.jp/cgi-bin/sogo.cgi?sid=&pj=640&action=newPlaceSite&site=S000080)

POLASIG1 1310 (+) AATAAA [S000080](https://sogo.dna.affrc.go.jp/cgi-bin/sogo.cgi?sid=&pj=640&action=newPlaceSite&site=S000080)

POLASIG2 726 (-) AATTAAA [S000081](https://sogo.dna.affrc.go.jp/cgi-bin/sogo.cgi?sid=&pj=640&action=newPlaceSite&site=S000081)

POLASIG2 835 (+) AATTAAA [S000081](https://sogo.dna.affrc.go.jp/cgi-bin/sogo.cgi?sid=&pj=640&action=newPlaceSite&site=S000081)

POLASIG2 952 (+) AATTAAA [S000081](https://sogo.dna.affrc.go.jp/cgi-bin/sogo.cgi?sid=&pj=640&action=newPlaceSite&site=S000081)

POLASIG2 1350 (+) AATTAAA [S000081](https://sogo.dna.affrc.go.jp/cgi-bin/sogo.cgi?sid=&pj=640&action=newPlaceSite&site=S000081)

POLASIG2 1379 (+) AATTAAA [S000081](https://sogo.dna.affrc.go.jp/cgi-bin/sogo.cgi?sid=&pj=640&action=newPlaceSite&site=S000081)

POLASIG2 1397 (+) AATTAAA [S000081](https://sogo.dna.affrc.go.jp/cgi-bin/sogo.cgi?sid=&pj=640&action=newPlaceSite&site=S000081)

POLASIG2 1502 (-) AATTAAA [S000081](https://sogo.dna.affrc.go.jp/cgi-bin/sogo.cgi?sid=&pj=640&action=newPlaceSite&site=S000081)

POLASIG3 51 (+) AATAAT [S000088](https://sogo.dna.affrc.go.jp/cgi-bin/sogo.cgi?sid=&pj=640&action=newPlaceSite&site=S000088)

POLASIG3 438 (-) AATAAT [S000088](https://sogo.dna.affrc.go.jp/cgi-bin/sogo.cgi?sid=&pj=640&action=newPlaceSite&site=S000088)

POLASIG3 663 (-) AATAAT [S000088](https://sogo.dna.affrc.go.jp/cgi-bin/sogo.cgi?sid=&pj=640&action=newPlaceSite&site=S000088)

POLASIG3 682 (+) AATAAT [S000088](https://sogo.dna.affrc.go.jp/cgi-bin/sogo.cgi?sid=&pj=640&action=newPlaceSite&site=S000088)

POLASIG3 718 (-) AATAAT [S000088](https://sogo.dna.affrc.go.jp/cgi-bin/sogo.cgi?sid=&pj=640&action=newPlaceSite&site=S000088)

POLASIG3 753 (-) AATAAT [S000088](https://sogo.dna.affrc.go.jp/cgi-bin/sogo.cgi?sid=&pj=640&action=newPlaceSite&site=S000088)

POLASIG3 848 (-) AATAAT [S000088](https://sogo.dna.affrc.go.jp/cgi-bin/sogo.cgi?sid=&pj=640&action=newPlaceSite&site=S000088)

POLASIG3 885 (+) AATAAT [S000088](https://sogo.dna.affrc.go.jp/cgi-bin/sogo.cgi?sid=&pj=640&action=newPlaceSite&site=S000088)

POLASIG3 1234 (-) AATAAT [S000088](https://sogo.dna.affrc.go.jp/cgi-bin/sogo.cgi?sid=&pj=640&action=newPlaceSite&site=S000088)

POLASIG3 1367 (+) AATAAT [S000088](https://sogo.dna.affrc.go.jp/cgi-bin/sogo.cgi?sid=&pj=640&action=newPlaceSite&site=S000088)

POLASIG3 1376 (+) AATAAT [S000088](https://sogo.dna.affrc.go.jp/cgi-bin/sogo.cgi?sid=&pj=640&action=newPlaceSite&site=S000088)

POLASIG3 1510 (+) AATAAT [S000088](https://sogo.dna.affrc.go.jp/cgi-bin/sogo.cgi?sid=&pj=640&action=newPlaceSite&site=S000088)

POLASIG3 1540 (-) AATAAT [S000088](https://sogo.dna.affrc.go.jp/cgi-bin/sogo.cgi?sid=&pj=640&action=newPlaceSite&site=S000088)

POLLEN1LELAT52 224 (+) AGAAA [S000245](https://sogo.dna.affrc.go.jp/cgi-bin/sogo.cgi?sid=&pj=640&action=newPlaceSite&site=S000245)

POLLEN1LELAT52 451 (-) AGAAA [S000245](https://sogo.dna.affrc.go.jp/cgi-bin/sogo.cgi?sid=&pj=640&action=newPlaceSite&site=S000245)

POLLEN1LELAT52 509 (-) AGAAA [S000245](https://sogo.dna.affrc.go.jp/cgi-bin/sogo.cgi?sid=&pj=640&action=newPlaceSite&site=S000245)

POLLEN1LELAT52 535 (+) AGAAA [S000245](https://sogo.dna.affrc.go.jp/cgi-bin/sogo.cgi?sid=&pj=640&action=newPlaceSite&site=S000245)

POLLEN1LELAT52 1048 (+) AGAAA [S000245](https://sogo.dna.affrc.go.jp/cgi-bin/sogo.cgi?sid=&pj=640&action=newPlaceSite&site=S000245)

POLLEN1LELAT52 1076 (+) AGAAA [S000245](https://sogo.dna.affrc.go.jp/cgi-bin/sogo.cgi?sid=&pj=640&action=newPlaceSite&site=S000245)

POLLEN1LELAT52 1143 (-) AGAAA [S000245](https://sogo.dna.affrc.go.jp/cgi-bin/sogo.cgi?sid=&pj=640&action=newPlaceSite&site=S000245)

POLLEN1LELAT52 1301 (+) AGAAA [S000245](https://sogo.dna.affrc.go.jp/cgi-bin/sogo.cgi?sid=&pj=640&action=newPlaceSite&site=S000245)

POLLEN1LELAT52 1442 (+) AGAAA [S000245](https://sogo.dna.affrc.go.jp/cgi-bin/sogo.cgi?sid=&pj=640&action=newPlaceSite&site=S000245)

PREATPRODH 230 (-) ACTCAT [S000450](https://sogo.dna.affrc.go.jp/cgi-bin/sogo.cgi?sid=&pj=640&action=newPlaceSite&site=S000450)

PREATPRODH 305 (+) ACTCAT [S000450](https://sogo.dna.affrc.go.jp/cgi-bin/sogo.cgi?sid=&pj=640&action=newPlaceSite&site=S000450)

PYRIMIDINEBOXOSRAMY1A 480 (-) CCTTTT [S000259](https://sogo.dna.affrc.go.jp/cgi-bin/sogo.cgi?sid=&pj=640&action=newPlaceSite&site=S000259)

QELEMENTZMZM13 1273 (-) AGGTCA [S000254](https://sogo.dna.affrc.go.jp/cgi-bin/sogo.cgi?sid=&pj=640&action=newPlaceSite&site=S000254)

RBCSCONSENSUS 1195 (+) AATCCAA [S000127](https://sogo.dna.affrc.go.jp/cgi-bin/sogo.cgi?sid=&pj=640&action=newPlaceSite&site=S000127)

REALPHALGLHCB21 517 (+) AACCAA [S000362](https://sogo.dna.affrc.go.jp/cgi-bin/sogo.cgi?sid=&pj=640&action=newPlaceSite&site=S000362)

REALPHALGLHCB21 964 (+) AACCAA [S000362](https://sogo.dna.affrc.go.jp/cgi-bin/sogo.cgi?sid=&pj=640&action=newPlaceSite&site=S000362)

RHERPATEXPA7 554 (-) KCACGW [S000512](https://sogo.dna.affrc.go.jp/cgi-bin/sogo.cgi?sid=&pj=640&action=newPlaceSite&site=S000512)

ROOTMOTIFTAPOX1 67 (-) ATATT [S000098](https://sogo.dna.affrc.go.jp/cgi-bin/sogo.cgi?sid=&pj=640&action=newPlaceSite&site=S000098)

ROOTMOTIFTAPOX1 68 (+) ATATT [S000098](https://sogo.dna.affrc.go.jp/cgi-bin/sogo.cgi?sid=&pj=640&action=newPlaceSite&site=S000098)

ROOTMOTIFTAPOX1 193 (+) ATATT [S000098](https://sogo.dna.affrc.go.jp/cgi-bin/sogo.cgi?sid=&pj=640&action=newPlaceSite&site=S000098)

ROOTMOTIFTAPOX1 867 (+) ATATT [S000098](https://sogo.dna.affrc.go.jp/cgi-bin/sogo.cgi?sid=&pj=640&action=newPlaceSite&site=S000098)

ROOTMOTIFTAPOX1 914 (+) ATATT [S000098](https://sogo.dna.affrc.go.jp/cgi-bin/sogo.cgi?sid=&pj=640&action=newPlaceSite&site=S000098)

ROOTMOTIFTAPOX1 938 (+) ATATT [S000098](https://sogo.dna.affrc.go.jp/cgi-bin/sogo.cgi?sid=&pj=640&action=newPlaceSite&site=S000098)

ROOTMOTIFTAPOX1 1022 (+) ATATT [S000098](https://sogo.dna.affrc.go.jp/cgi-bin/sogo.cgi?sid=&pj=640&action=newPlaceSite&site=S000098)

ROOTMOTIFTAPOX1 1114 (-) ATATT [S000098](https://sogo.dna.affrc.go.jp/cgi-bin/sogo.cgi?sid=&pj=640&action=newPlaceSite&site=S000098)

ROOTMOTIFTAPOX1 1131 (+) ATATT [S000098](https://sogo.dna.affrc.go.jp/cgi-bin/sogo.cgi?sid=&pj=640&action=newPlaceSite&site=S000098)

ROOTMOTIFTAPOX1 1139 (+) ATATT [S000098](https://sogo.dna.affrc.go.jp/cgi-bin/sogo.cgi?sid=&pj=640&action=newPlaceSite&site=S000098)

ROOTMOTIFTAPOX1 1357 (-) ATATT [S000098](https://sogo.dna.affrc.go.jp/cgi-bin/sogo.cgi?sid=&pj=640&action=newPlaceSite&site=S000098)

ROOTMOTIFTAPOX1 1358 (+) ATATT [S000098](https://sogo.dna.affrc.go.jp/cgi-bin/sogo.cgi?sid=&pj=640&action=newPlaceSite&site=S000098)

ROOTMOTIFTAPOX1 1370 (-) ATATT [S000098](https://sogo.dna.affrc.go.jp/cgi-bin/sogo.cgi?sid=&pj=640&action=newPlaceSite&site=S000098)

ROOTMOTIFTAPOX1 1513 (-) ATATT [S000098](https://sogo.dna.affrc.go.jp/cgi-bin/sogo.cgi?sid=&pj=640&action=newPlaceSite&site=S000098)

ROOTMOTIFTAPOX1 1520 (-) ATATT [S000098](https://sogo.dna.affrc.go.jp/cgi-bin/sogo.cgi?sid=&pj=640&action=newPlaceSite&site=S000098)

ROOTMOTIFTAPOX1 1546 (+) ATATT [S000098](https://sogo.dna.affrc.go.jp/cgi-bin/sogo.cgi?sid=&pj=640&action=newPlaceSite&site=S000098)

S1FBOXSORPS1L21 281 (-) ATGGTA [S000223](https://sogo.dna.affrc.go.jp/cgi-bin/sogo.cgi?sid=&pj=640&action=newPlaceSite&site=S000223)

S1FBOXSORPS1L21 1090 (+) ATGGTA [S000223](https://sogo.dna.affrc.go.jp/cgi-bin/sogo.cgi?sid=&pj=640&action=newPlaceSite&site=S000223)

SEF1MOTIF 914 (+) ATATTTAWW [S000006](https://sogo.dna.affrc.go.jp/cgi-bin/sogo.cgi?sid=&pj=640&action=newPlaceSite&site=S000006)

SEF1MOTIF 938 (+) ATATTTAWW [S000006](https://sogo.dna.affrc.go.jp/cgi-bin/sogo.cgi?sid=&pj=640&action=newPlaceSite&site=S000006)

SEF1MOTIF 1131 (+) ATATTTAWW [S000006](https://sogo.dna.affrc.go.jp/cgi-bin/sogo.cgi?sid=&pj=640&action=newPlaceSite&site=S000006)

SEF3MOTIFGM 1449 (-) AACCCA [S000115](https://sogo.dna.affrc.go.jp/cgi-bin/sogo.cgi?sid=&pj=640&action=newPlaceSite&site=S000115)

SEF4MOTIFGM7S 195 (+) RTTTTTR [S000103](https://sogo.dna.affrc.go.jp/cgi-bin/sogo.cgi?sid=&pj=640&action=newPlaceSite&site=S000103)

SEF4MOTIFGM7S 831 (-) RTTTTTR [S000103](https://sogo.dna.affrc.go.jp/cgi-bin/sogo.cgi?sid=&pj=640&action=newPlaceSite&site=S000103)

SEF4MOTIFGM7S 851 (+) RTTTTTR [S000103](https://sogo.dna.affrc.go.jp/cgi-bin/sogo.cgi?sid=&pj=640&action=newPlaceSite&site=S000103)

SEF4MOTIFGM7S 881 (-) RTTTTTR [S000103](https://sogo.dna.affrc.go.jp/cgi-bin/sogo.cgi?sid=&pj=640&action=newPlaceSite&site=S000103)

SEF4MOTIFGM7S 889 (+) RTTTTTR [S000103](https://sogo.dna.affrc.go.jp/cgi-bin/sogo.cgi?sid=&pj=640&action=newPlaceSite&site=S000103)

SEF4MOTIFGM7S 894 (-) RTTTTTR [S000103](https://sogo.dna.affrc.go.jp/cgi-bin/sogo.cgi?sid=&pj=640&action=newPlaceSite&site=S000103)

SEF4MOTIFGM7S 990 (+) RTTTTTR [S000103](https://sogo.dna.affrc.go.jp/cgi-bin/sogo.cgi?sid=&pj=640&action=newPlaceSite&site=S000103)

SEF4MOTIFGM7S 1064 (-) RTTTTTR [S000103](https://sogo.dna.affrc.go.jp/cgi-bin/sogo.cgi?sid=&pj=640&action=newPlaceSite&site=S000103)

SEF4MOTIFGM7S 1306 (-) RTTTTTR [S000103](https://sogo.dna.affrc.go.jp/cgi-bin/sogo.cgi?sid=&pj=640&action=newPlaceSite&site=S000103)

SEF4MOTIFGM7S 1312 (-) RTTTTTR [S000103](https://sogo.dna.affrc.go.jp/cgi-bin/sogo.cgi?sid=&pj=640&action=newPlaceSite&site=S000103)

SEF4MOTIFGM7S 1333 (+) RTTTTTR [S000103](https://sogo.dna.affrc.go.jp/cgi-bin/sogo.cgi?sid=&pj=640&action=newPlaceSite&site=S000103)

SEF4MOTIFGM7S 1353 (-) RTTTTTR [S000103](https://sogo.dna.affrc.go.jp/cgi-bin/sogo.cgi?sid=&pj=640&action=newPlaceSite&site=S000103)

SITEIIATCYTC 300 (-) TGGGCY [S000474](https://sogo.dna.affrc.go.jp/cgi-bin/sogo.cgi?sid=&pj=640&action=newPlaceSite&site=S000474)

SORLIP2AT 299 (+) GGGCC [S000483](https://sogo.dna.affrc.go.jp/cgi-bin/sogo.cgi?sid=&pj=640&action=newPlaceSite&site=S000483)

SORLIP2AT 300 (-) GGGCC [S000483](https://sogo.dna.affrc.go.jp/cgi-bin/sogo.cgi?sid=&pj=640&action=newPlaceSite&site=S000483)

SORLREP3AT 606 (+) TGTATATAT [S000488](https://sogo.dna.affrc.go.jp/cgi-bin/sogo.cgi?sid=&pj=640&action=newPlaceSite&site=S000488)

SREATMSD 1170 (-) TTATCC [S000470](https://sogo.dna.affrc.go.jp/cgi-bin/sogo.cgi?sid=&pj=640&action=newPlaceSite&site=S000470)

SURE1STPAT21 221 (+) AATAGAAAA [S000186](https://sogo.dna.affrc.go.jp/cgi-bin/sogo.cgi?sid=&pj=640&action=newPlaceSite&site=S000186)

TAAAGSTKST1 7 (-) TAAAG [S000387](https://sogo.dna.affrc.go.jp/cgi-bin/sogo.cgi?sid=&pj=640&action=newPlaceSite&site=S000387)

TAAAGSTKST1 99 (-) TAAAG [S000387](https://sogo.dna.affrc.go.jp/cgi-bin/sogo.cgi?sid=&pj=640&action=newPlaceSite&site=S000387)

TAAAGSTKST1 701 (+) TAAAG [S000387](https://sogo.dna.affrc.go.jp/cgi-bin/sogo.cgi?sid=&pj=640&action=newPlaceSite&site=S000387)

TAAAGSTKST1 1059 (+) TAAAG [S000387](https://sogo.dna.affrc.go.jp/cgi-bin/sogo.cgi?sid=&pj=640&action=newPlaceSite&site=S000387)

TATABOX2 1133 (-) TATAAAT [S000109](https://sogo.dna.affrc.go.jp/cgi-bin/sogo.cgi?sid=&pj=640&action=newPlaceSite&site=S000109)

TATABOX3 665 (+) TATTAAT [S000110](https://sogo.dna.affrc.go.jp/cgi-bin/sogo.cgi?sid=&pj=640&action=newPlaceSite&site=S000110)

TATABOX3 720 (+) TATTAAT [S000110](https://sogo.dna.affrc.go.jp/cgi-bin/sogo.cgi?sid=&pj=640&action=newPlaceSite&site=S000110)

TATABOX3 1236 (+) TATTAAT [S000110](https://sogo.dna.affrc.go.jp/cgi-bin/sogo.cgi?sid=&pj=640&action=newPlaceSite&site=S000110)

TATABOX3 1237 (-) TATTAAT [S000110](https://sogo.dna.affrc.go.jp/cgi-bin/sogo.cgi?sid=&pj=640&action=newPlaceSite&site=S000110)

TATABOX3 1251 (+) TATTAAT [S000110](https://sogo.dna.affrc.go.jp/cgi-bin/sogo.cgi?sid=&pj=640&action=newPlaceSite&site=S000110)

TATABOX4 780 (-) TATATAA [S000111](https://sogo.dna.affrc.go.jp/cgi-bin/sogo.cgi?sid=&pj=640&action=newPlaceSite&site=S000111)

TATABOX4 1135 (-) TATATAA [S000111](https://sogo.dna.affrc.go.jp/cgi-bin/sogo.cgi?sid=&pj=640&action=newPlaceSite&site=S000111)

TATABOX5 412 (-) TTATTT [S000203](https://sogo.dna.affrc.go.jp/cgi-bin/sogo.cgi?sid=&pj=640&action=newPlaceSite&site=S000203)

TATABOX5 439 (+) TTATTT [S000203](https://sogo.dna.affrc.go.jp/cgi-bin/sogo.cgi?sid=&pj=640&action=newPlaceSite&site=S000203)

TATABOX5 527 (-) TTATTT [S000203](https://sogo.dna.affrc.go.jp/cgi-bin/sogo.cgi?sid=&pj=640&action=newPlaceSite&site=S000203)

TATABOX5 799 (-) TTATTT [S000203](https://sogo.dna.affrc.go.jp/cgi-bin/sogo.cgi?sid=&pj=640&action=newPlaceSite&site=S000203)

TATABOX5 804 (-) TTATTT [S000203](https://sogo.dna.affrc.go.jp/cgi-bin/sogo.cgi?sid=&pj=640&action=newPlaceSite&site=S000203)

TATABOX5 849 (+) TTATTT [S000203](https://sogo.dna.affrc.go.jp/cgi-bin/sogo.cgi?sid=&pj=640&action=newPlaceSite&site=S000203)

TATABOX5 884 (-) TTATTT [S000203](https://sogo.dna.affrc.go.jp/cgi-bin/sogo.cgi?sid=&pj=640&action=newPlaceSite&site=S000203)

TATABOX5 918 (+) TTATTT [S000203](https://sogo.dna.affrc.go.jp/cgi-bin/sogo.cgi?sid=&pj=640&action=newPlaceSite&site=S000203)

TATABOX5 994 (+) TTATTT [S000203](https://sogo.dna.affrc.go.jp/cgi-bin/sogo.cgi?sid=&pj=640&action=newPlaceSite&site=S000203)

TATABOX5 1005 (+) TTATTT [S000203](https://sogo.dna.affrc.go.jp/cgi-bin/sogo.cgi?sid=&pj=640&action=newPlaceSite&site=S000203)

TATABOX5 1009 (+) TTATTT [S000203](https://sogo.dna.affrc.go.jp/cgi-bin/sogo.cgi?sid=&pj=640&action=newPlaceSite&site=S000203)

TATABOX5 1013 (+) TTATTT [S000203](https://sogo.dna.affrc.go.jp/cgi-bin/sogo.cgi?sid=&pj=640&action=newPlaceSite&site=S000203)

TATABOX5 1303 (-) TTATTT [S000203](https://sogo.dna.affrc.go.jp/cgi-bin/sogo.cgi?sid=&pj=640&action=newPlaceSite&site=S000203)

TATABOX5 1309 (-) TTATTT [S000203](https://sogo.dna.affrc.go.jp/cgi-bin/sogo.cgi?sid=&pj=640&action=newPlaceSite&site=S000203)

TATABOX5 1315 (-) TTATTT [S000203](https://sogo.dna.affrc.go.jp/cgi-bin/sogo.cgi?sid=&pj=640&action=newPlaceSite&site=S000203)

TATABOX5 1366 (-) TTATTT [S000203](https://sogo.dna.affrc.go.jp/cgi-bin/sogo.cgi?sid=&pj=640&action=newPlaceSite&site=S000203)

TATABOX5 1470 (+) TTATTT [S000203](https://sogo.dna.affrc.go.jp/cgi-bin/sogo.cgi?sid=&pj=640&action=newPlaceSite&site=S000203)

TATABOXOSPAL 939 (+) TATTTAA [S000400](https://sogo.dna.affrc.go.jp/cgi-bin/sogo.cgi?sid=&pj=640&action=newPlaceSite&site=S000400)

TATAPVTRNALEU 1134 (+) TTTATATA [S000340](https://sogo.dna.affrc.go.jp/cgi-bin/sogo.cgi?sid=&pj=640&action=newPlaceSite&site=S000340)

TATCCAOSAMY 464 (+) TATCCA [S000403](https://sogo.dna.affrc.go.jp/cgi-bin/sogo.cgi?sid=&pj=640&action=newPlaceSite&site=S000403)

TATCCAOSAMY 1169 (-) TATCCA [S000403](https://sogo.dna.affrc.go.jp/cgi-bin/sogo.cgi?sid=&pj=640&action=newPlaceSite&site=S000403)

TATCCAYMOTIFOSRAMY3D 1168 (-) TATCCAY [S000256](https://sogo.dna.affrc.go.jp/cgi-bin/sogo.cgi?sid=&pj=640&action=newPlaceSite&site=S000256)

TBOXATGAPB 16 (-) ACTTTG [S000383](https://sogo.dna.affrc.go.jp/cgi-bin/sogo.cgi?sid=&pj=640&action=newPlaceSite&site=S000383)

TGTCACACMCUCUMISIN 420 (+) TGTCACA [S000422](https://sogo.dna.affrc.go.jp/cgi-bin/sogo.cgi?sid=&pj=640&action=newPlaceSite&site=S000422)

UP2ATMSD 104 (+) AAACCCTA [S000472](https://sogo.dna.affrc.go.jp/cgi-bin/sogo.cgi?sid=&pj=640&action=newPlaceSite&site=S000472)

WBOXATNPR1 405 (-) TTGAC [S000390](https://sogo.dna.affrc.go.jp/cgi-bin/sogo.cgi?sid=&pj=640&action=newPlaceSite&site=S000390)

WBOXATNPR1 826 (+) TTGAC [S000390](https://sogo.dna.affrc.go.jp/cgi-bin/sogo.cgi?sid=&pj=640&action=newPlaceSite&site=S000390)

WBOXHVISO1 179 (-) TGACT [S000442](https://sogo.dna.affrc.go.jp/cgi-bin/sogo.cgi?sid=&pj=640&action=newPlaceSite&site=S000442)

WBOXHVISO1 233 (-) TGACT [S000442](https://sogo.dna.affrc.go.jp/cgi-bin/sogo.cgi?sid=&pj=640&action=newPlaceSite&site=S000442)

WBOXHVISO1 404 (-) TGACT [S000442](https://sogo.dna.affrc.go.jp/cgi-bin/sogo.cgi?sid=&pj=640&action=newPlaceSite&site=S000442)

WBOXHVISO1 827 (+) TGACT [S000442](https://sogo.dna.affrc.go.jp/cgi-bin/sogo.cgi?sid=&pj=640&action=newPlaceSite&site=S000442)

WBOXNTCHN48 233 (-) CTGACY [S000508](https://sogo.dna.affrc.go.jp/cgi-bin/sogo.cgi?sid=&pj=640&action=newPlaceSite&site=S000508)

WBOXNTERF3 179 (-) TGACY [S000457](https://sogo.dna.affrc.go.jp/cgi-bin/sogo.cgi?sid=&pj=640&action=newPlaceSite&site=S000457)

WBOXNTERF3 233 (-) TGACY [S000457](https://sogo.dna.affrc.go.jp/cgi-bin/sogo.cgi?sid=&pj=640&action=newPlaceSite&site=S000457)

WBOXNTERF3 404 (-) TGACY [S000457](https://sogo.dna.affrc.go.jp/cgi-bin/sogo.cgi?sid=&pj=640&action=newPlaceSite&site=S000457)

WBOXNTERF3 827 (+) TGACY [S000457](https://sogo.dna.affrc.go.jp/cgi-bin/sogo.cgi?sid=&pj=640&action=newPlaceSite&site=S000457)

WBOXNTERF3 1273 (+) TGACY [S000457](https://sogo.dna.affrc.go.jp/cgi-bin/sogo.cgi?sid=&pj=640&action=newPlaceSite&site=S000457)

WRKY71OS 78 (-) TGAC [S000447](https://sogo.dna.affrc.go.jp/cgi-bin/sogo.cgi?sid=&pj=640&action=newPlaceSite&site=S000447)

WRKY71OS 180 (-) TGAC [S000447](https://sogo.dna.affrc.go.jp/cgi-bin/sogo.cgi?sid=&pj=640&action=newPlaceSite&site=S000447)

WRKY71OS 234 (-) TGAC [S000447](https://sogo.dna.affrc.go.jp/cgi-bin/sogo.cgi?sid=&pj=640&action=newPlaceSite&site=S000447)

WRKY71OS 405 (-) TGAC [S000447](https://sogo.dna.affrc.go.jp/cgi-bin/sogo.cgi?sid=&pj=640&action=newPlaceSite&site=S000447)

WRKY71OS 421 (-) TGAC [S000447](https://sogo.dna.affrc.go.jp/cgi-bin/sogo.cgi?sid=&pj=640&action=newPlaceSite&site=S000447)

WRKY71OS 827 (+) TGAC [S000447](https://sogo.dna.affrc.go.jp/cgi-bin/sogo.cgi?sid=&pj=640&action=newPlaceSite&site=S000447)

WRKY71OS 1273 (+) TGAC [S000447](https://sogo.dna.affrc.go.jp/cgi-bin/sogo.cgi?sid=&pj=640&action=newPlaceSite&site=S000447)
